# The distribution of haemoglobin C and its prevalence in newborns in Africa

**DOI:** 10.1038/srep01671

**Published:** 2013-04-17

**Authors:** Frédéric B. Piel, Rosalind E. Howes, Anand P. Patil, Oscar A. Nyangiri, Peter W. Gething, Samir Bhatt, Thomas N. Williams, David J. Weatherall, Simon I. Hay

**Affiliations:** 1 Spatial Ecology and Epidemiology Group, Tinbergen Building, Department of Zoology, University of Oxford, South Parks Road, Oxford OX1 3PS, United Kingdom; 2 Evolutionary Ecology of Infectious Disease Group, Tinbergen Building, Department of Zoology, University of Oxford, South Parks Road, Oxford OX1 3PS, United Kingdom; 3 Kenya Medical Research Institute/Wellcome Trust Programme, Centre for Geographic Medicine Research-Coast, PO Box 230, Kilifi District Hospital, Kilifi, Kenya; 4 Department of Medicine, Imperial College, St Mary's Hospital, Praed Street, London W21NY, United Kingdom; 5 Weatherall Institute of Molecular Medicine, John Radcliffe Hospital, Oxford OX3 9DS, United Kingdom

## Abstract

Haemoglobin C (HbC) is one of the commonest structural haemoglobin variants in human populations. Although HbC causes mild clinical complications, its diagnosis and genetic counselling are important to prevent inheritance with other haemoglobinopathies. Little is known about its contemporary distribution and the number of newborns affected. We assembled a global database of population surveys. We then used a Bayesian geostatistical model to create maps of HbC frequency across Africa and paired our predictions with high-resolution demographics to calculate heterozygous (AC) and homozygous (CC) newborn estimates and their associated uncertainty. Data were too sparse outside Africa for this methodology to be applied. The highest frequencies were found in West Africa but HbC was commonly found in other parts of the continent. The expected annual numbers of AC and CC newborns in Africa were 672,117 (interquartile range (IQR): 642,116-705,163) and 28,703 (IQR: 26,027-31,958), respectively. These numbers are about two times previous estimates.

Haemoglobin C (HbC) is a structural variant of normal haemoglobin (HbA) caused by an amino acid substitution at position 6 of the β-globin chain (β6Glu-Lys)^1^. It is one of the most prevalent abnormal haemoglobin mutations globally alongside haemoglobin S, which occurs at the same position (HbS; β6Glu-Val), and haemoglobin E (HbE, β26Glu-Lys). In HbC heterozygote individuals (AC), this trait is asymptomatic. Homozygosity (CC) causes clinically mild haemolytic anaemia, due to the reduced solubility of the red blood cells which can lead to crystal formation^2^. HbC is mainly of clinical significance when inherited in combination with HbS (sickle-haemoglobin C disease), causing chronic haemolytic anaemia and intermittent sickle cell crises, slightly less severe or frequent than in homozygous HbS patients (SS), and when co-inherited with β-thalassaemia (haemoglobin C-β thalassaemia), causing moderate haemolytic anaemia with splenomegaly^3^.

HbC allele frequencies above 15% have been described in West African populations^4^. As for HbS, the selection pressure resulting from malaria protection has been suggested to explain the high prevalence of this polymorphism in a number of populations (commonly referred to as the malaria hypothesis)^5^,^6^. It has been found that HbC provides near full protection against *Plasmodium falciparum* malaria in homozygous (CC) individuals and intermediate protection in heterozygous (AC) individuals^7^. Although these advantages (milder clinical severity and protection from severe and fatal *Plasmodium falciparum* malaria in both AC and CC individuals) could suggest that HbC has better fitness than HbS^8^,^9^, until the recent waves of human migration in the last few centuries, its distribution was limited to a much smaller geographic area than that of HbS^5^.

HbC is now widespread^10^,^11^,^12^,^13^, and it is widely assumed that HbC expanded to its current distribution from a unique origin in West Africa^14^,^15^,^16^, although an independent origin in southeast Asia has been suggested^17^,^18^. The current distribution of HbC is poorly documented^4^,^19^, yet this information is necessary to assess its contribution to the increasing public health and economic burden of the haemoglobinopathies^20^. Here, as part of our efforts to create an open access online database of selected inherited blood disorders and polymorphisms^5^,^21^,^22^,^23^, we have reviewed the published literature and assembled representative population survey data on HbC allele frequencies at the global scale. Following careful inclusion criteria and georeferencing of these data, this database formed the evidence-base for a Bayesian model-based geostatistical (MBG) framework^24^,^25^ which we developed to predict a continuous map of the distribution of HbC across Africa. Pairing these predictions with high resolution population data and national crude birth rates allowed the expected numbers of newborns affected annually by HbC trait (AC) and disease (CC) to be estimated.

## Results

### Database

**Figure 1:**
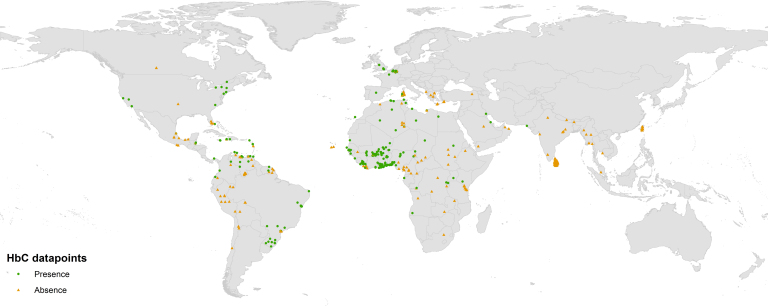
Global distribution of surveys on HbC. Green dots and orange triangles indicate surveys which found HbC to be present and absent from the population sample respectively. Created with ESRI ArcGIS 10.1.

Our searches identified 174 data sources (listed in Supplementary Information) with HbC data which allowed calculation of an allele frequency for representative population samples at specific locations. These included data for 445 spatially unique locations (Figure 1). The total number of individuals tested was 7,540,983. Sample sizes ranged from four individuals to 3,212,374. The mean sample size was 16,946 individuals. Some 82% of the population samples tested fewer than 1,000 individuals. Although 45% of the population surveys were conducted on the African continent, these represented only 5% of the total number of individuals examined. Our searches revealed that about half (51%) of the total 1,992 references from the online searches on HbC found has been published after 1985, the publication year of Livingstone's latest database (Supplementary Figure S1)^4^. About 60% of the surveys used for the present study pre-dated 1985. Amongst our 445 datapoints, an absence of HbC was reported in 48% of them. Few surveys (n = 7) indicating null frequencies within West Africa (Figure 1) have been published. Allele frequencies above 20% were observed in the eastern (29%) and western (24%) parts of Burkina Faso. Apart from one survey in southern Ghana, frequencies above 10% were only observed across Burkina Faso and the adjacent northern parts of Ghana, Togo and Benin (32 surveys). Although HbC has been found in other parts of Africa (including Angola, Kenya, Egypt and Algeria), none of the surveys conducted in southern Cameroon (seven surveys) or southern Chad (three surveys) reported its presence. In North America, HbC was found both on the West and East coast of the United States (18 surveys), but was absent from eight of nine surveys in Mexico. In South America, HbC was identified in most surveys conducted in Brazil (33 surveys) and was commonly observed (14 out of 38 surveys) in Columbia, Venezuela and French Guiana. It was not observed in Peru, Bolivia or Chile (15 surveys). Ten of the 13 surveys conducted in the Caribbean islands reported the presence of HbC. In Europe, HbC was observed in capital cities (London, Paris, Madrid, and Brussels) as well as in parts of Sardinia, but not in Greece or Albania. In the Middle East, surveys conducted on the eastern coast of Saudi Arabia, in eastern Iraq and along the Pakistani coast each reported a few cases. In Asia, none of the population surveys, including the micro-mapping survey conducted in Sri Lanka, found HbC. No surveys were available from Oceania.

### Map

**Figure 2:**
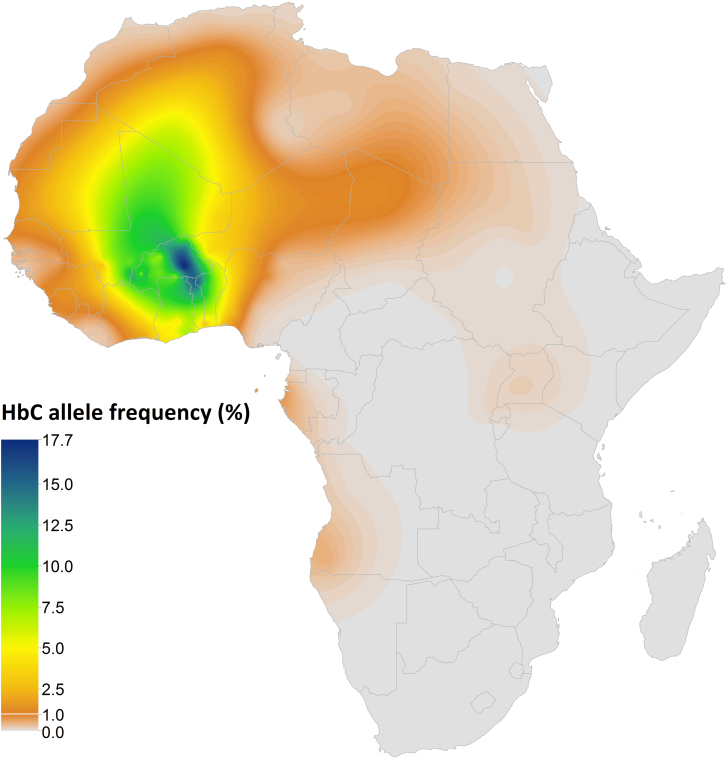
Summary map of HbC predicted allele frequency in Africa. Raster map (5 km × 5 km) of HbC allele frequency (posterior mean) generated by a Bayesian model-based geostatistical framework. Created with ESRI ArcGIS 10.1.

**Figure 3:**
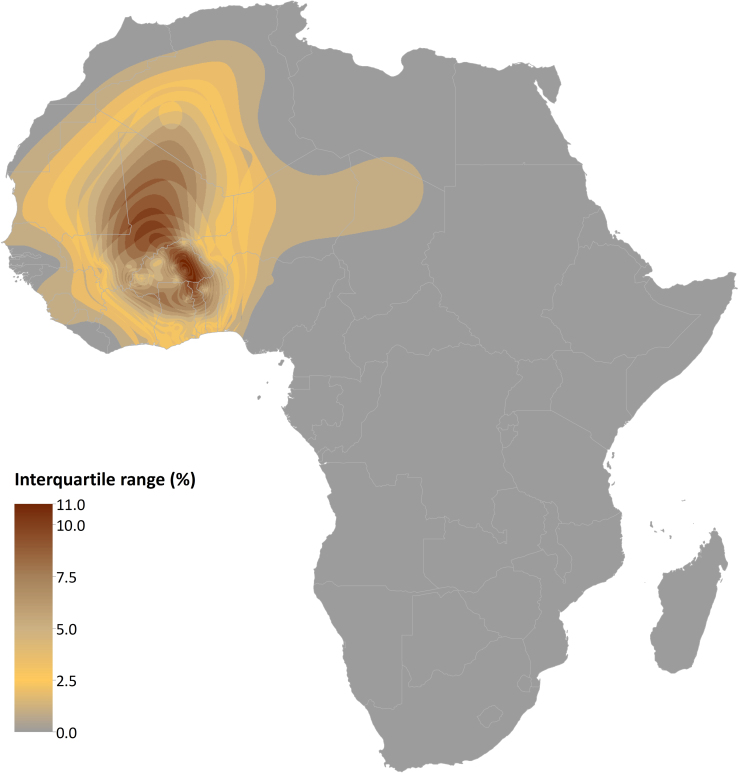
Uncertainty map in HbC predicted allele frequency in Africa. Interquartile range (50% probability) of the per-pixel predicted allele frequency. Created with ESRI ArcGIS 10.1.

Our continuous map predicted HbC allele frequencies across Africa. The predicted posterior mean is presented in Figure 2. The maximum of the predicted posterior median HbC allele frequency was 16.0% (interquartile range (IQR): 12.0%–21.0%) in the eastern part of Burkina Faso. Median frequencies above 12.5% were predicted around that area, as well as in north-eastern Ghana, northern Togo and north-western Benin. We predicted median frequencies above 7.5% in western Burkina Faso (up to 11.0% (IQR: 8.0%–14.0%), remaining parts of northern Ghana and Benin, as well as across most of Mali, eastern Mauritania and southern Algeria. Median frequencies reached up to 5.0% in most other parts of western Africa, despite pockets of low frequencies (e.g. in Sierra Leone and Guinea-Bissau) and a sharp longitudinal decrease across Nigeria. The model also predicted a corridor of mean frequencies of about 1.0% between West Africa and Egypt, based on the finding of HbC in the few surveys conducted in these areas of low population density. Patches of median frequencies below 1% were predicted in Gabon, eastern Angola, and Uganda. The uncertainty associated with these predictions is shown in Figure 3. The IQR distribution reflects the distribution and heterogeneity of the data. It reaches values up to 11.0% (IQR: 9.0%–20.0%) in eastern Burkina Faso. The IQR is mostly above 5.0% across Mali and northern Ghana, Togo and Benin where very few surveys were available.

### National and regional estimates of affected newborns

We estimated that, in 2010, in Africa, 672,117 (IQR: 642,116-705,163) and 28,703 babies (IQR: 26,027-31,958) were born with the AC and CC genotypes respectively (Table 1). At the national scale, 56% of the AC newborns were expected to be from Burkina Faso (131,454 [IQR: 117,825-146,173]), Ghana (98,153 [IQR: 87,225-110,939]) and Nigeria (IQR: 148,423 [112,961-197,818]), and 76% of the CC newborns in these three countries (Burkina Faso: 9,592 [IQR: 7,258-13,259]); Ghana: 4,707 [IQR: 3,601-6,546]) and Nigeria: 3,099 [IQR: 1,822-5,948]) and Mali (4,354 [IQR: 2,257-9,952]).

### Validation statistics

The mean error, mean absolute error and root mean square (RMS) error associated with our allele frequency predictions were 0.012 + 0.026, 0.019 + 0.027, and 0.026 + 0.037, respectively. The overall bias of the predictions is thus very small, while their accuracy and precision can be considered as good. The Monte Carlo standard errors (SE) associated with the areal estimates at regional and national levels respectively are shown in Supplementary Table S1.

## Discussion

It is expected that the global economic burden of the haemoglobinopathies on public health will increase over the coming decades^20^. In order to assess this burden and to track spatial and temporal changes, it is crucial to have a good knowledge of the distribution and number of individuals affected by these disorders. The map of surveys on HbC provides a summary of currently available data and highlights areas where further research is needed. The predicted allele frequency maps reflect the contemporary distribution of this disorder in Africa. The newborn estimates give us a more precise idea of the public health importance of HbC. Each result is discussed in detail below.

The originally confined distribution of HbC was described as early as the mid-1950s^26^ - only a few years after the first identification of this particular haemoglobin variant^1^. Nevertheless, cartographic refinements have been nearly absent since then. In 1967, Frank B. Livingstone published impressively detailed maps of abnormal haemoglobins, which included HbC, but he did not publish equivalent maps in the updated version of his database in 1985^27^. Further, these maps were discontinuous, both spatially (i.e. mapping data as points but not predicting at unobserved locations) and quantitatively (i.e. using a categorical allele frequency scale). In 1994, Cavalli-Sforza *et al* created a continuous allele frequency map of HbC as part of their suite of genetic maps^28^. The aim of the present study was to incorporate the additional data collected over the last decades and the technological improvements in mapping and modelling methods^27^,^28^,^29^, allowing to provide precision metrics for the first time.

The distribution of datapoints somehow summarises our current knowledge of HbC. Although most of the surveys were conducted in West Africa, where the highest frequencies are expected, their sample sizes are usually limited. The presence of HbC in surveys in Brazil, the United States and European capitals for which data were available (e.g. London and Paris) reflect the presence of immigrants from West Africa in these communities. More detailed data from Belgium suggest that HbC carriers might also be identified in smaller cities (Boemer, *pers. com.*) but no surveys focussed on non-urban areas where HbC is likely to be absent due to lower levels of admixture. Because of the small number of surveys available in the Middle East and of the possible misidentification of HbC and HbE with commonly used electrophoretic methods^3^, the eastern limit of the distribution of HbC is unclear.

According to our input data and predicted maps, HbC reaches its highest predicted frequencies in the western part of Burkina Faso. Our model suggests that high frequencies (□ 7.5%) might extend across Burkina Faso, the northern parts of Ghana, Togo and Benin, and across Mali, eastern Mauritania and the southern part of Algeria. In the absence of any data from this area this is only informed by the common presence of HbC in northern Africa. Conducting population surveys in this area, as well as in Côte d'Ivoire and northern Ghana, would help refine knowledge of the extent of the distribution of high HbC allele frequencies in West Africa.

It is usually considered that the HbS mutation occurred at least twice in human history, once in Africa and once in Asia^30^,^31^,^32^,^33^; although several haplotypes have been identified for the HbC mutation, it is assumed to have a single origin in western Africa^15^,^34^. A recent case study conducted in Thailand suggested that one local haplotype might indicate an independent non-African origin of HbC^18^. The small number of cases identified so far, the absence of HbC in population surveys included in our database for India and Southeast Asia, and the absence of HbC in recent unpublished studies carried in Cambodia, Malaysia and South China (Fucharoen, *pers. com.*) tend to suggest that this haplotype would have a very limited distribution and low frequency. Furthermore, not a single case of HbC was identified during the micro-mapping work conducted in Sri Lanka (Weatherall, *pers. com.*). Further investigation into the hypothesis of an independent HbC mutation in Southeast Asia and its fitness in the presence of thalassaemias and haemoglobin E would provide a valuable contribution to our understanding of epistatic interactions between haemoglobinopathies^35^,^36^.

There is strong evidence for the protective effect of HbS against clinical *Plasmodium falciparum* malaria^37^. It is usually assumed that HbC also protects against malaria, but to a much lesser extent than HbS as reflected by its relatively limited original distribution^8^. An apparent inverse correlation between HbS and HbC allele frequencies in West Africa has been described^8^,^33^,^38^. The map presented here represents the contemporary distribution of HbC within Africa which because of human migration over the last few centuries is less useful than a map of pre-migration frequencies for investigating such a correlation^5^. Such maps and investigations are planned in future applications of this work. Furthermore, there is some evidence suggesting that other genes might also affect the level of malaria protection conveyed by HbC^39^.

Global, regional and national estimates of population affected represent important tools to assess the status of a particular disease, to follow its changes over space and time, and to guide associated public health policy. In 2008, Modell and Darlison published various estimates, including the proportion of pregnant women with AC and the number of CC conceptions, and derived service indicators for the haemoglobin disorders^19^. Here, we overcome several methodological limitations in order to provide updated newborn estimates. First, we performed online searches covering the 1950–2010 period to include recent data and selected data based on strict inclusion criteria to exclude non-representative surveys. Second, each survey was georeferenced as precisely as possible, enabling better representation of spatial heterogeneity. Third, we used high-resolution population distribution data to relate these heterogeneities with the distribution of human populations. Finally, we calculated our predictions within a Bayesian MBG framework, which allowed calculation of the precision associated with our estimates.

Using 2003 UN demographics, Modell and Darlison estimated a global total of 14,719 CC newborns including 14,227 (97%) in the AFRO region (Table 1). Their estimates were conservative (i.e. minimum figures) and estimates for countries where no HbC data were available were set to zero (e.g. Kenya or the Democratic Republic of the Congo). No estimates were published for the AC newborns and the precision of the estimates published was unknown. Our regional estimate for CC newborns in 2010 is about twofold higher, at 28,703 newborns (×1.8 for the 25%-quartile estimate and ×2.2 for the 75%-quartile estimate). This large difference is mostly due to higher predictions in West African countries. Because of high heterogeneity in HbC allele frequency, extrapolating an average allele frequency to a national population can lead to an underestimation (or overestimation) of the number of individuals affected. In Burkina Faso, most of the survey data come from the western part where frequencies are comparatively lower than in the eastern part of the country. Similarly, there is very little data from the northern parts of Ghana, Togo and Benin – countries in which HbC allele frequencies tend to gradually increase across a latitudinal gradient (Ohene-Frempong, *pers. com.*). Not accounting for the distribution of the surveys within West African countries or their population distribution (i.e. high population density in areas of low HbC frequency and low population density in areas of high frequency will produce dramatically different estimates compared to the reverse situation) could therefore result in underestimating the number of newborns affected.

Although HbC causes only relatively mild clinical complications in AC and CC individuals, a good knowledge of its distribution and allele frequencies represents a useful tool to better assess its contribution, through compound individuals with HbSC disease or HbC/β-thalassaemia, to the global burden of the haemoglobinopathies^20^,^40^. A bespoke multi-allelic model, ideally using an age-correction based on the mortality of SC individuals and accounting for deviation from Hardy-Weinberg assumptions^41^,^42^, would be required to estimate the number of SC newborns within a similar framework. The availability of the map and estimates presented here for HbC, alongside similar products developed for HbS^22^, will facilitate such a study. The current lack of an updated database on the thalassaemias makes the calculation of the number of HbC/β-thalassaemia compound newborns currently difficult. These various limitations will be the focus of future work.

Globally, our database suggests that human migrations are probably the main drivers shaping the contemporary distribution of the haemoglobinopathies, exerting a greater influence than positive selection driven by protection against malaria, which seems to have been the main factor in the past^5^,^33^. Although selection is likely to still influence the distribution of haemoglobinopathies in malarious regions, this change could contribute to shaping their future distributions, particularly in the context of epidemiological transition^20^ and malaria elimination^43^ efforts. The work presented here could not fully reflect those changes in the distribution of HbC due to the relative paucity of data outside of Africa. Nevertheless, our map provides a unique picture of the current distribution of HbC in Africa, while our estimates suggest that the annual number of AC and CC newborns might have been largely underestimated previously. In the long term, additional data will allow creating a global contemporary map and calculating global estimates and the development of a multi-allelic model would allow the calculation of similar estimates for SC and C-βThal compound individuals, which have a greater impact on clinical burden. This forms part of our plans for further work on the haemoglobinopathies.

## Methods

**Figure 4:**
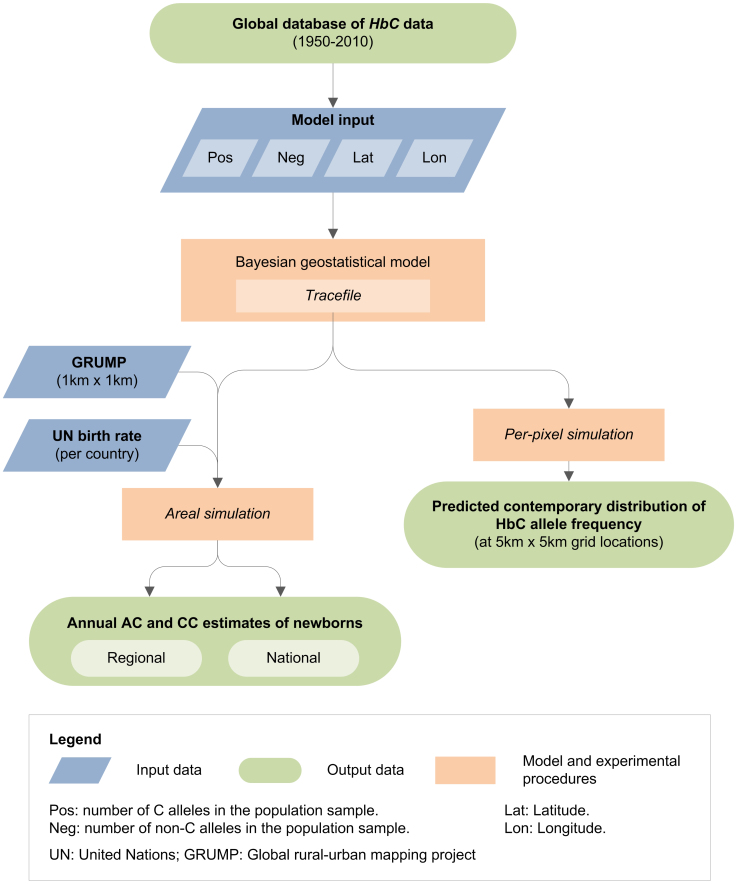
Schematic overview of the approach. Blue diamonds describe input data. Orange boxes denote models and experimental procedures. Green rods indicate output data. Created with Microsoft Office Visio 2007.

A schematic overview of the methods used is provided as Figure 4. The methodology is briefly described below. Further details are available in the Supplementary Information.

### Data sources

To identify publications with HbC allele frequency data, we undertook a comprehensive online data search using PubMed^44^, ISI Web of Knowledge^45^, and Scopus^46^ bibliographic databases. The following keywords were used: ‘haemoglobin C’ and ‘hemoglobin C’. Searches performed on May 3, 2011 returned 1,275, 558 and 1,827 references in PubMed, ISI Web of Science and Scopus respectively. After duplicate removal, we identified 1,992 unique references which were then reviewed according to inclusion criteria, the main ones being that: i) the source included primary data on HbC frequency; ii) the population samples were representative of the local communities (data from targeted screening or selected population samples, such as Afro-Americans, were excluded) and iii) the survey location could be georeferenced precisely^5^. Additional data from unpublished sources fulfilling these criteria (particularly from the MalariaGEN Consortium)^47^ were also included in the global database. When several surveys conducted at the same location met the inclusion criteria, only the most representative one (based on a combination of criteria including the year of the survey, the sample size and the diagnostic method) was used. The list of data sources used is shown in the Supplementary Information and the database is freely available online (http://www.map.ox.ac.uk).

### Bayesian model-based geostatistical (MBG) framework

Numbers of A (*neg*) and C (*pos*) alleles, based on the number of AA, AC and CC individuals found in each population survey conducted on the African continent were used as input to the model alongside the surveys' geographic coordinates (latitude and longitude). For studies reporting the absence of any haemoglobin variants, all individuals were considered as AA. The model generated two distinct types of output across Africa: estimates of HbC allele frequency for every 5 × 5 km pixel (i.e. estimates at point locations), and estimates of AC and CC newborns within each African country (i.e. estimates over areal units). In both cases, the full posterior predictive distribution (PPD)^24^, was generated for the target quantity using 500,000 Markov chain Monte Carlo (MCMC) iterations^48^. A complete description of the model is given in the Supplementary Information.

### Predicted distribution map in Africa

We assumed Hardy-Weinberg equilibrium for the calculation of AC and CC individuals from the HbC predicted allele frequency^5^,^24^. The mean and interquartile range (IQR; interval between the 25% and 75% percentiles of the PPD) maps were used to summarise the predictions and their associated uncertainty, respectively, for each pixel in African countries.

### Estimates of newborns affected in Africa

The AC and CC predicted frequencies were weighted by i) high resolution (1 × 1 km) population data from the 2010-adjusted beta version of the Global Rural Urban Mapping Project (GRUMP)^49^, and ii) national crude birth rates for 2010, derived from the 2010 revision online population database of the United Nations (UN) world population prospects^50^ (see Supplementary Information). To allow assessment of uncertainty measures associated with these aggregated population numbers, estimates were calculated using sampling from the whole predictive distributions of areal integrals (not just the mean summary map) within the area considered^22^,^24^,^51^. Areal estimates were calculated independently for the regional and national predictions. Summary estimates presented here include the median and IQR of the population predictions.

### Model validation

Validation metrics were calculated by comparing the observed allele frequency for a 10% random hold-out sample of the African subset with the prediction output created from the remaining 90% of the African data^5^. The validation metrics were summarised by i) the mean error, which indicates the average distance between the actual data points and the predicted values; ii) the mean absolute error, which measures the average magnitude of the errors in the predicted values; and iii) the root mean square (RMS) error^29^. These errors provide a measure of the model's overall bias, overall accuracy and overall precision, respectively. In order to calculate the Monte Carlo standard error (SE)^52^ associated with the newborn estimates, the areal calculations were repeated ten times at each scale (see Supplementary Information).

## Author Contributions

F.B.P. assembled the data, conceived the study and wrote the first draft of the manuscript with S.I.H.; O.A.N. and R.E.H. helped assemble and abstract the data; A.P.P. and P.W.G. conceived and helped implement the modelling and all computational tasks. All authors contributed to the revision of the final manuscript. The authors declare no competing financial interests. This work forms part of the output of the Malaria Atlas Project (MAP, http://www.map.ox.ac.uk), principally funded by the Wellcome Trust, U.K.

## Supplementary Material

Supplementary Information Supplementary Information

## Figures and Tables

**Table 1:** National demographic indicators and estimates of HbC allele frequency and newborns affected within the AFRO WHO region

Country/Region	Total population (in thousands)	CBR^1^	Surveys	HbC AF (IQR^2^)	AC newborns/yr (IQR^2^)	CC newborns/yr (IQR^2^)	M&D^3^
Algeria	35,423	0.0192	15	0.003 (0.002–0.005)	3,439 (1,839–7,119)	46 (15–159)	72
Angola	18,994	0.0399	1	0.001 (0.000–0.006)	1,510 (257–9,539)	4 (0–102)	0
Benin	9,219	0.0383	9	0.064 (0.046–0.090)	41,915 (32,952–52,400)	1,892 (1,188–3,200)	746
Botswana	1,977	0.0229	0	0.000 (0.000–0.000)	0 (0–11)	0 (0–0)	0
Burkina Faso	16,250	0.0424	42	0.130 (0.108–0.158)	131,454 (117,825–146,173)	9,592 (7,258–13,259)	3,730
Burundi	8,519	0.0333	1	0.000 (0.000–0.002)	132 (31–578)	0 (0–1)	0
Cameroon	19,957	0.0349	11	0.001 (0.000–0.001)	400 (146–1,127)	0 (0–3)	5
Cape Verde	513	0.0199	2	0.000 (0.000–0.001)	1 (0–10)	0 (0–0)	0
Central African Republic	4,506	0.0345	0	0.000 (0.000–0.001)	27 (4–187)	0 (0–0)	0
Chad	11,509	0.0434	5	0.002 (0.001–0.004)	1,282 (390–4,183)	7 (1–58)	6
Comoros	691	0.0357	0	0.000 (0.000–0.000)	0 (0–1)	0 (0–0)	0
Congo	3,760	0.0346	1	0.001 (0.000–0.003)	112 (21–898)	0 (0–6)	0
Congo, the Democratic Republic of the	67,829	0.0421	4	0.001 (0.000–0.001)	1,813 (594–6,213)	2 (0–30)	0
Côte d'Ivoire	21,571	0.0330	3	0.028 (0.017–0.049)	42,277 (27,050–64,339)	1,244 (576–2,922)	935
Equatorial Guinea	693	0.0359	0	0.001 (0.000–0.004)	38 (10–177)	0 (0–1)	0
Eritrea	5,204	0.0344	0	0.000 (0.000–0.002)	20 (1–379)	0 (0–1)	0
Ethiopia	84,996	0.0300	1	0.000 (0.000–0.002)	470 (36–6,754)	0 (0–21)	0
Gabon	1,501	0.0270	1	0.004 (0.001–0.009)	299 (81–838)	1 (0–9)	0
Gambia	1,751	0.0369	12	0.005 (0.003–0.008)	609 (302–1,138)	2 (1–9)	8
Ghana	24,339	0.0303	18	0.074 (0.061–0.090)	98,153 (87,225–110,939)	4,707 (3,601–6,546)	2,501
Guinea	10,324	0.0376	1	0.013 (0.007–0.023)	11,186 (5,931–19,970)	162 (49–497)	0
Guinea-Bissau	1,647	0.0374	1	0.003 (0.001–0.006)	303 (108–815)	1 (0–6)	1
Kenya	40,835	0.0369	1	0.001 (0.000–0.003)	1,048 (170–7,501)	1 (0–29)	0
Lesotho	2,064	0.0271	0	0.000 (0.000–0.000)	0 (0–2)	0 (0–0)	0
Liberia	4,102	0.0376	12	0.004 (0.002–0.007)	1,275 (620–2,476)	6 (2–21)	4
Madagascar	20,146	0.0346	0	0.000 (0.000–0.000)	0 (0–11)	0 (0–0)	0
Malawi	15,690	0.0445	0	0.000 (0.000–0.000)	4 (0–97)	0 (0–0)	0
Mali	13,362	0.0450	7	0.078 (0.049–0.128)	79,506 (58,011–106,112)	4,354 (2,257–9,952)	1,616
Mauritania	3,359	0.0327	1	0.023 (0.009–0.066)	5,309 (2,136–11,459)	145 (24–808)	18
Mauritius	1,297	0.0125	0	0.000 (0.000–0.000)	0 (0–0)	0 (0–0)	0
Mozambique	23,418	0.0363	0	0.000 (0.000–0.000)	2 (0–68)	0 (0–0)	0
Namibia	2,212	0.0252	0	0.001 (0.000–0.003)	32 (3–378)	0 (0–2)	0
Niger	15,885	0.0477	3	0.025 (0.015–0.049)	40,670 (24,006–69,159)	1,196 (527–3,068)	734
Nigeria	158,255	0.0393	16	0.011 (0.008–0.015)	148,423 (112,961–197,818)	3,099 (1,822–5,948)	3,278
Rwanda	10,277	0.0406	1	0.001 (0.000–0.002)	446 (129–1,554)	0 (0–3)	0
Sao Tome and Principe	165	0.0299	0	0.004 (0.001–0.026)	36 (3–305)	0 (0–6)	1
Senegal	12,866	0.0359	3	0.007 (0.004–0.014)	7,326 (3,508–14,548)	56 (13–230)	64
Sierra Leone	5,837	0.0365	0	0.009 (0.004–0.022)	4,508 (1,575–11,076)	40 (6–228)	64
South Africa	50,523	0.0205	1	0.000 (0.000–0.000)	1 (0–61)	0 (0–0)	0
Swaziland	1,195	0.0287	0	0.000 (0.000–0.000)	0 (0–1)	0 (0–0)	0
Tanzania, United Republic of	45,028	0.0410	13	0.000 (0.000–0.001)	558 (123–3,033)	0 (0–8)	0
Togo	6,774	0.0310	3	0.082 (0.059–0.112)	29,093 (23,448–35,050)	1,594 (989–2,702)	446
Uganda	33,798	0.0439	1	0.001 (0.000–0.004)	2,721 (618–12,531)	4 (0–69)	0
Zambia	13,254	0.0465	2	0.000 (0.000–0.000)	23 (1–221)	0 (0–0)	0
Zimbabwe	12,645	0.0287	0	0.000 (0.000–0.000)	2 (0–40)	0 (0–0)	0
AFRO region	888,817	0.0357	198	0.011 (0.011–0.012)	672,117 (642,116–705,163)	28,703 (26,027–31,958)	14,227

^1^CBR: Crude birth rate.

^2^IQR: interquartile range.

^3^M&D: Modell & Darlison, 2008.
