# Supplementary material for: The distribution of haemoglobin C and its prevalence in newborns in Africa
Source: Sci Rep. 2013 Apr 17;3:1671. doi: 10.1038/srep01671 (PMC3628164; doi:10.1038/srep01671)
Supplement: Supplementary Information [file srep01671-s1.pdf]

Supplementary Information

The distribution of haemoglobin C and its prevalence in newborns in Africa

Frédéric B. Piel[1,2*], Rosalind E. Howes[1], Anand P. Patil[1], Oscar A. Nyangiri[3], Peter W. Gething[1], Samir Bhatt[1], Thomas N. Williams[3,4], David J. Weatherall[5] & Simon I. Hay[1]

1 Spatial Ecology and Epidemiology Group, Tinbergen Building, Department of Zoology, University of Oxford, South Parks Road, Oxford OX1 3PS, United Kingdom.
2 Evolutionary Ecology of Infectious Disease Group, Tinbergen Building, Department of Zoology, University of Oxford, South Parks Road, Oxford OX1 3PS, United Kingdom.
3 Kenya Medical Research Institute/Wellcome Trust Programme, Centre for Geographic Medicine Research-Coast, PO Box 230, Kilifi District Hospital, Kilifi, Kenya.
4 Nuffield Department of Clinical Medicine, University of Oxford, Oxford OX3 7LJ, United Kingdom.
5 Weatherall Institute of Molecular Medicine, John Radcliffe Hospital, Oxford OX3 9DS, United Kingdom.

This Supplementary Information includes:

Supplementary Figures:
- Supplementary Figure S1: Temporal distribution of the published literature on HbC.
- Supplementary Figure S2: Map of the distribution of the HbC data points used as thinned and holdout validation datasets, within Africa.
- Supplementary Figure S3: Validation plots

Supplementary Tables:
- Supplementary Table S1: Monte Carlo standard errors associated with the HbC national and regional areal predictions.
- Supplementary Table S2: MCMC output parameter values.

Supplementary Methods:
- Inclusion criteria
- Georeferencing protocol
- Bayesian model-based geostatistical framework
    a. Model
    b. Prior
    c. Likelihood
    d. Flexible link function and empirical Bayesian analysis
    e. Empirical Bayesian approach to fitting the polynomial coefficients
    f. Prior predictive constraint
    g. Fitting the model
    h. Mapping procedure
    i. Mean vs. median
    j. Model validation
    k. Demographic data
    l. Areal predictions
    m. Monte Carlo standard errors
- References

Supplementary References:
- Supplementary References. Sources from which data points were identified.

Supplementary Figures

Supplementary Figure S1. Temporal distribution of the published literature on HbC. PubMed: n=1,273; ISI: n= 554 and Scopus: n=1,820. All combined after duplicate removal: n=1,963.

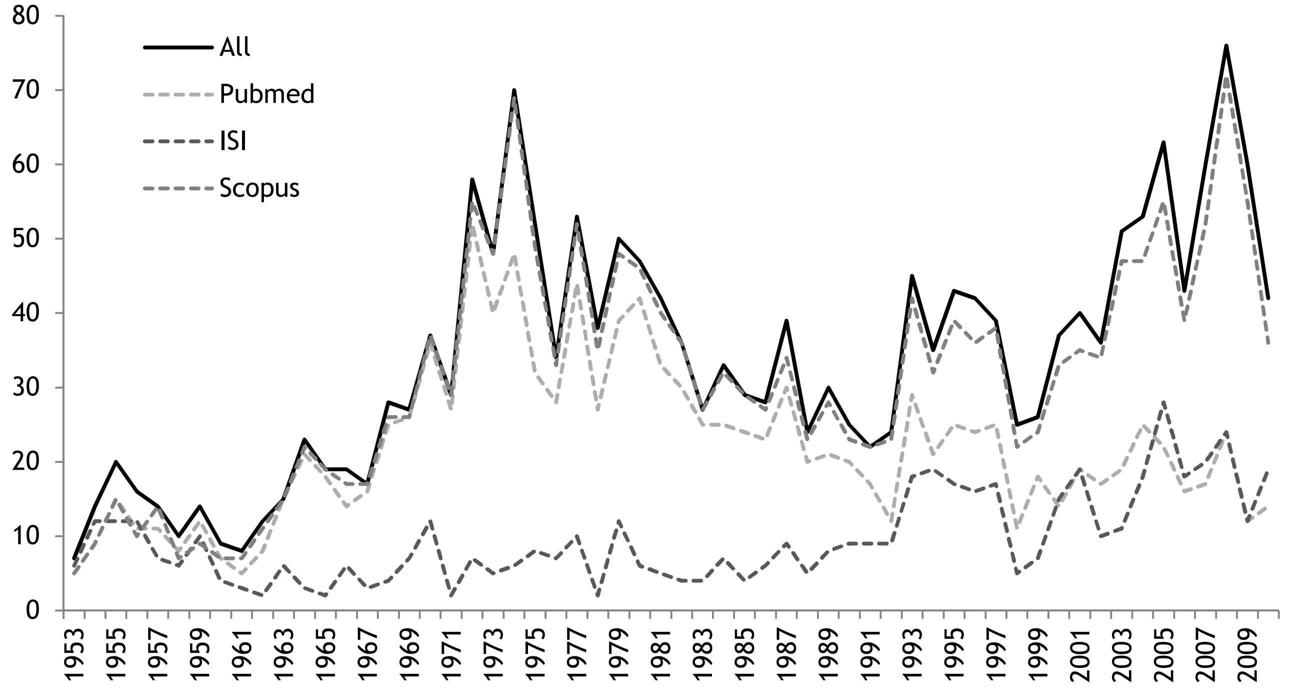

Supplementary Figure S2. Map of the distribution of the randomly selected subsample of the HbC data points used for the model validation, in Africa. Blue dots: thinned subset (90%); red circles: holdout subset (10%).

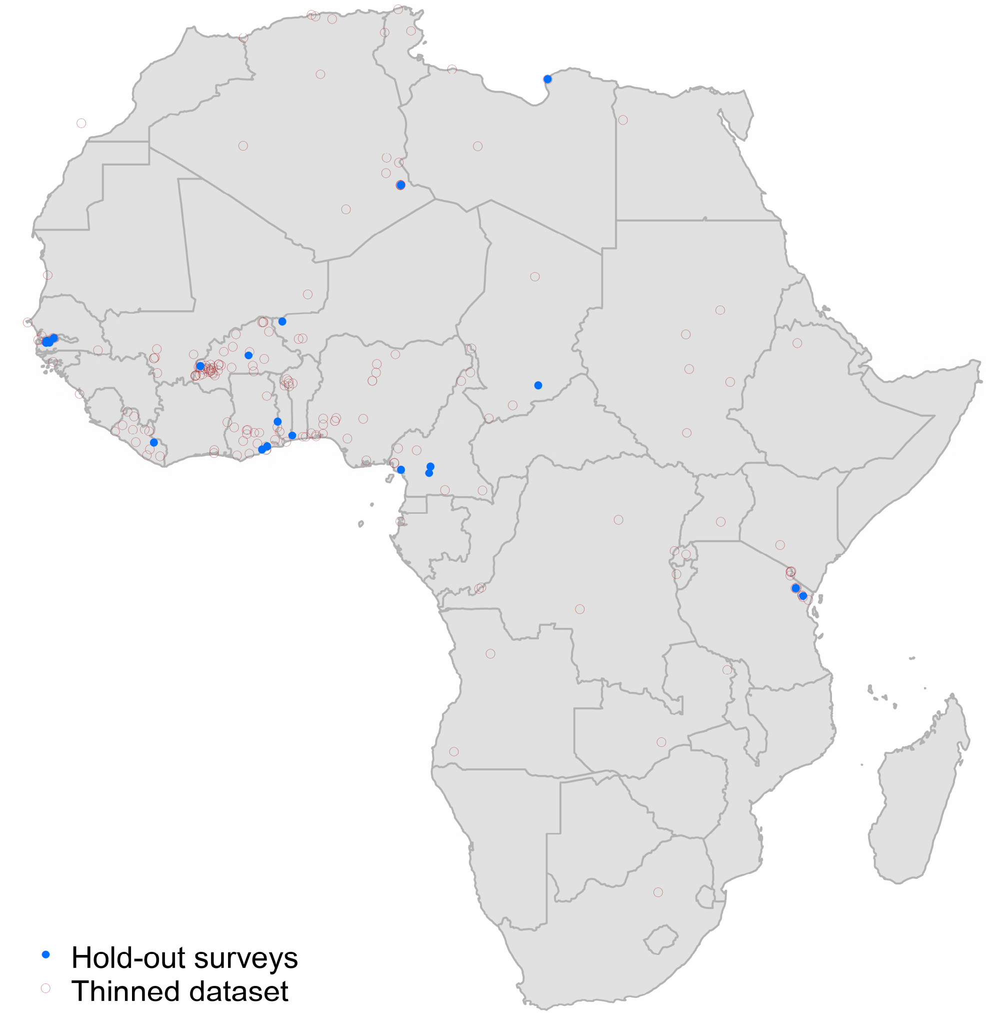

Supplementary Figure S3. Validation plots comparing the HbC prediction with the observed allele frequency for the data points from the hold-out subset of the data (n=20). A. Scatter plot of the observed vs. predicted allele frequency; B. Plot of the observed vs. predicted quantiles.

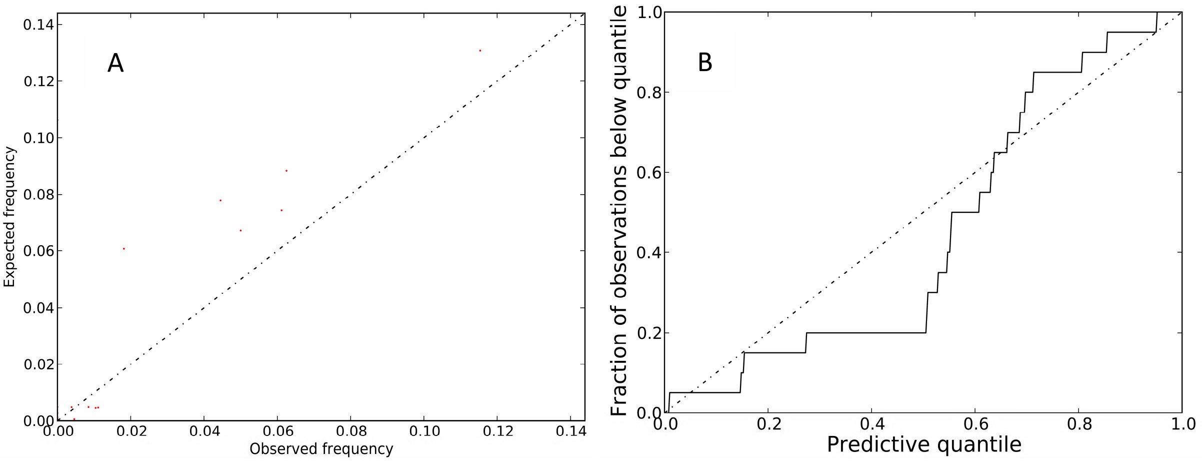

Supplementary Table S1: National areal prediction summaries and Monte Carlo standard errors (SE) for AC and CC newborn estimates within the AFRO WHO region

	HbC heterozygote newborns (AC)								HbC homozygote newborns (CC)							
**COUNTRY**	**Mean**	***SE***	**Median**	***SE***	**Q25%**	***SE***	**Q75%**	***SE***	**Mean**	***SE***	**Median**	***SE***	**Q25%**	***SE***	**Q75%**	***SE***
Algeria	3,837	*187*	3,439	*217*	1,839	*179*	7,119	*101*	62	*3*	46	*4*	15	*1*	159	*12*
Angola	2,770	*139*	1,510	*210*	257	*79*	9,539	*208*	23	*2*	4	*0*	0	*0*	102	*10*
Benin	40,503	*945*	41,915	*1,031*	32,952	*1,145*	52,400	*504*	2,004	*15*	1,892	*21*	1,188	*47*	3,200	*166*
Botswana	3	*1*	0	*0*	0	*0*	11	*4*	0	*0*	0	*0*	0	*0*	0	*0*
Burkina Faso	133,533	*1,648*	131,454	*1,499*	117,825	*156*	146,173	*3,689*	9,830	*760*	9,592	*673*	7,258	*234*	13,259	*1,613*
Burundi	192	*54*	132	*48*	31	*12*	578	*120*	0	*0*	0	*0*	0	*0*	1	*0*
Cameroon	481	*116*	400	*113*	146	*53*	1,127	*189*	1	*0*	0	*0*	0	*0*	3	*0*
Cape Verde	3	*1*	1	*0*	0	*0*	10	*3*	0	*0*	0	*0*	0	*0*	0	*0*
Central African Republic	53	*14*	27	*10*	4	*2*	187	*39*	0	*0*	0	*0*	0	*0*	0	*0*
Chad	1,620	*95*	1,282	*115*	390	*78*	4,183	*89*	16	*1*	7	*0*	1	*0*	58	*5*
Comoros	1	*0*	0	*0*	0	*0*	1	*1*	0	*0*	0	*0*	0	*0*	0	*0*
Congo	267	*28*	112	*33*	21	*9*	898	*25*	2	*0*	0	*0*	0	*0*	6	*1*
Côte d'Ivoire	39,830	*1,449*	42,277	*1,584*	27,050	*929*	64,339	*1,566*	1,434	*41*	1,244	*68*	576	*45*	2,922	*67*
Democratic Republic of the Congo	2,354	*424*	1,813	*436*	594	*208*	6,213	*584*	8	*0*	2	*0*	0	*0*	30	*2*
Djibouti	5	*1*	0	*0*	0	*0*	18	*6*	0	*0*	0	*0*	0	*0*	0	*0*
Egypt	2,817	*207*	578	*200*	29	*12*	12,441	*632*	28	*3*	0	*0*	0	*0*	64	*7*
Equatorial Guinea	59	*7*	38	*8*	10	*3*	177	*9*	0	*0*	0	*0*	0	*0*	1	*0*
Eritrea	99	*18*	20	*8*	1	*0*	379	*73*	0	*0*	0	*0*	0	*0*	1	*0*
Ethiopia	1,647	*242*	470	*173*	36	*14*	6,754	*812*	10	*1*	0	*0*	0	*0*	21	*1*
Gabon	361	*6*	299	*12*	81	*16*	838	*27*	3	*0*	1	*0*	0	*0*	9	*1*
Ghana	98,589	*2,238*	98,153	*2,309*	87,225	*2,844*	110,939	*1,366*	4,843	*29*	4,707	*71*	3,601	*157*	6,546	*239*
Guinea	11,459	*397*	11,186	*385*	5,931	*73*	19,970	*842*	206	*16*	162	*15*	49	*4*	497	*34*
Guinea-Bissau	331	*23*	303	*29*	108	*24*	815	*9*	2	*0*	1	*0*	0	*0*	6	*0*
Kenya	2,095	*301*	1,048	*308*	170	*68*	7,501	*548*	8	*0*	1	*0*	0	*0*	29	*2*
Lesotho	1	*0*	0	*0*	0	*0*	2	*1*	0	*0*	0	*0*	0	*0*	0	*0*
Liberia	1,344	*40*	1,275	*51*	620	*65*	2,476	*29*	8	*0*	6	*0*	2	*0*	21	*2*
Libyan Arab Jamahiriya	562	*51*	480	*58*	173	*40*	1,313	*34*	3	*0*	1	*0*	0	*0*	10	*1*
Madagascar	5	*2*	0	*0*	0	*0*	11	*5*	0	*0*	0	*0*	0	*0*	0	*0*
Malawi	24	*8*	4	*2*	0	*0*	97	*37*	0	*0*	0	*0*	0	*0*	0	*0*
Mali	92,567	*715*	79,506	*1,147*	58,011	*2,173*	106,112	*2,318*	4,999	*318*	4,354	*105*	2,257	*88*	9,952	*1,472*
Mauritania	6,046	*139*	5,309	*166*	2,136	*44*	11,459	*168*	247	*15*	145	*6*	24	*2*	808	*95*
Mauritius	0	*0*	0	*0*	0	*0*	0	*0*	0	*0*	0	*0*	0	*0*	0	*0*
Mayotte	0	*0*	0	*0*	0	*0*	0	*0*	0	*0*	0	*0*	0	*0*	0	*0*
Morocco	3,979	*97*	1,878	*197*	202	*62*	14,815	*600*	56	*4*	7	*0*	0	*0*	240	*24*
Mozambique	17	*6*	2	*1*	0	*0*	68	*27*	0	*0*	0	*0*	0	*0*	0	*0*
Namibia	96	*8*	32	*9*	3	*1*	378	*14*	1	*0*	0	*0*	0	*0*	2	*0*

**COUNTRY**	**Mean**	***SE***	**Median**	***SE***	**Q25%**	***SE***	**Q75%**	***SE***	**Mean**	***SE***	**Median**	***SE***	**Q25%**	***SE***	**Q75%**	***SE***
Niger	41,833	*1,456*	40,670	*1,567*	24,006	*779*	69,159	*1,946*	1,425	*57*	1,196	*77*	527	*44*	3,068	*43*
Nigeria	151,207	*4,645*	148,423	*4,797*	112,961	*3,061*	197,818	*6,137*	3,407	*227*	3,099	*258*	1,822	*172*	5,948	*188*
Réunion	0	*0*	0	*0*	0	*0*	0	*0*	0	*0*	0	*0*	0	*0*	0	*0*
Rwanda	616	*135*	446	*135*	129	*52*	1,554	*209*	1	*0*	0	*0*	0	*0*	3	*0*
Sao Tome and Principe	80	*1*	36	*3*	3	*1*	305	*13*	1	*0*	0	*0*	0	*0*	6	*1*
Senegal	7,639	*95*	7,326	*51*	3,508	*196*	14,548	*525*	81	*6*	56	*4*	13	*0*	230	*20*
Sierra Leone	5,247	*140*	4,508	*102*	1,575	*97*	11,076	*531*	70	*6*	40	*3*	6	*0*	228	*21*
Somalia	63	*13*	11	*5*	0	*0*	244	*67*	0	*0*	0	*0*	0	*0*	0	*0*
South Africa	15	*5*	1	*0*	0	*0*	61	*24*	0	*0*	0	*0*	0	*0*	0	*0*
Sudan	2,818	*319*	2,074	*368*	543	*179*	7,433	*310*	16	*1*	4	*0*	0	*0*	62	*5*
Swaziland	0	*0*	0	*0*	0	*0*	1	*0*	0	*0*	0	*0*	0	*0*	0	*0*
United Republic of Tanzania	951	*186*	558	*177*	123	*49*	3,033	*361*	2	*0*	0	*0*	0	*0*	8	*0*
Gambia	642	*19*	609	*24*	302	*34*	1,138	*18*	3	*0*	2	*0*	1	*0*	9	*1*
Togo	25,936	*325*	29,093	*366*	23,448	*613*	35,050	*156*	1,685	*71*	1,594	*41*	989	*31*	2,702	*282*
Tunisia	487	*64*	390	*72*	136	*42*	1,140	*59*	2	*0*	1	*0*	0	*0*	6	*0*
Uganda	4,097	*458*	2,721	*549*	618	*221*	12,531	*364*	17	*1*	4	*1*	0	*0*	69	*4*
Western Sahara	151	*1*	84	*3*	13	*3*	376	*18*	2	*0*	1	*0*	0	*0*	10	*1*
Zambia	57	*17*	23	*9*	1	*0*	221	*63*	0	*0*	0	*0*	0	*0*	0	*0*
Zimbabwe	10	*4*	2	*1*	0	*0*	40	*16*	0	*0*	0	*0*	0	*0*	0	*0*
**AFRO region**	**675,185**	***7,336***	**672,117**	***7,290***	**642,116**	***7,535***	**705,163**	***7,656***	**29,385**	***889***	**28,703**	***717***	**26,027**	***382***	**31,958**	***1,224***

Supplementary Table S2. MCMC output parameter values. Summary statistics presented are the mean and median values, standard deviation (std), interquartile range (IQR) and 95% Bayesian credible interval (95 BCI). The scale parameter is measured in units of earth radii. Values are presented to two significant figures.

Parameter	Symbol	Mean	Median	Std	IQR	95 BCI
Nugget variance	V	0.114	0.109	0.044	0.047	0.146
Amplitude (or partial sill)	ϕ	2.574	2.541	0.322	0.347	1.210
Scale (or range)	θ	0.490	0.493	0.010	0.012	0.037

Supplementary Methods

Inclusion criteria

Only population samples representative of the local communities were included. Detailed data given for all the ethnic groups found in the area (based on the information provided in the original source) were recorded as separate entries but entered as a single population survey in the model. Surveys targeting only specific ethnic groups (e.g. African Americans in North America) were excluded as in most cases the proportion of that particular ethnic group in the general population was unknown, as well as the allele frequency in the other ethnic groups present at the sampling site. Surveys focusing on hospital patients (e.g. minor ailments, fever or malaria) were also excluded, as they may represent biased estimates of the frequency in the general population.

When possible, authors were contacted for additional information concerning their studies in order to obtain missing information necessary to assess the quality of their results. Because of the possible misidentification of HbC and HbE with commonly used electrophoretic methods[1], only studies in which the identification of HbC appeared unambiguous were included.

No constraints were placed on sample size as, in a model-based geostatistical (MBG) framework (see below), there is no rational for excluding small sample sizes. This is because, on the one hand the model weighs the information content of each survey in accordance with a binomial sampling model, therefore down-weighting the information from very small samples, and on the other hand the uncertainty in relation to the sample size is explicitly modelled by this technique[2]. Nevertheless, case reports were excluded from this study as they did not match our criterion for representativeness of the local communities.

The number of normal (A or *neg*) and abnormal (C or *pos*) alleles observed was used as input for the model. For example, in a sample of N individuals tested in which n_{AA}, n_{AC}, n_{CC} were found to be AA, AC and CC individuals respectively, HbA and HbC allele frequencies are:

$$p = \frac{(2 * n_{AA}) + n_{AC}}{2N}$$

$$q = \frac{(2 * n_{CC}) + n_{AC}}{2N}$$

We assumed all populations to be at Hardy-Weinberg equilibrium (HWE)[3,4]. Although assuming random mating is inaccurate in most communities in northern Africa due to high consanguinity levels[5], , only scarce data on this factor was available in the data sources used for the present study and there is currently no consistent database allowing to quantify these factors regionally or globally.

Georeferencing

The geographic location of each survey was determined as precisely as possible using a georeferencing protocol adapted from Guerra *et al.*[6]. Author descriptions of survey sites were used to locate the sampling sites. Geographic coordinates (in decimal degrees, WGS84) were identified in various global gazetteers including the Encarta Reference Library 2007 (Microsoft Corporation, Redmond, WA, USA), Geonames (National Geospatial-Intelligence Agency. http://geonames.nga.mil/ggmagaz/) and Global Gazetteer Version 2.2 (Falling Rain Genomics Inc. http://www.fallingrain.com/world/index.html). Surveys were categorised according to the area that they represented: points (≤10km^2), wide areas (>10 and ≤25km^2), and small (>25 and ≤100km^2) or large polygons (>100km^2). Polygons were digitised and centroids calculated in GIS software (ArcView 3.2 and ArcMap 10.0, ESRI Inc., Redlands, CA, USA). A similar method was applied to surveys which could only be georeferenced to the district (admin2 unit) level. Surveys reported only to province (admin1 unit) or country (admin0 unit) level were considered to lack sufficient geographical specificity and were thus excluded. The geographic coordinates (latitude and longitude) were used as input in the model.

Bayesian model-based geostatistical framework

a. Model

In this section, we describe our Bayesian spatial model for the HbC allele frequency surface $(.)$ in Africa. *C* takes as its argument an arbitrary location on the Earth's surface within this continent. The posterior $[C]$ induces a posterior $[CC]$ for the HbC disease frequency surface $CC(.)$ since, using the Hardy-Weinberg[3,4] assumption that an individual's two copies of each allele are chosen independently from a gene pool, $CC(x) = C(x)^2$. We computed summaries of $[CC(x)]$, such as the mean $E(CC(x))$ and the variance $Var(CC(x))$, at each location x, to produce the maps related to haemoglobin C disease frequency in newborns.

The model differs from the model employed by Piel *et al.*[7] because, while conducting the analysis for the current paper, we diagnosed a lack of fit in our previous model that did not have a substantial effect on the main summaries of interest of the posterior for the allele frequency, but did cause serious errors in the posterior for homozygotes $[CC]$. This point is discussed further below.

The national-level disease burden $D(A)$ in nation A can be computed from CC, the birth rate b_A in *A* and the population density surface *N* using the areal integral:

$$D(A) = \int_A b_A N(x) CC(x) da$$

This is a deterministic transformation of CC and therefore of C, so theoretically the posterior $[C]$ induces the posterior $[D(A)]$. However, for reasons discussed by Patil *et al.*[8], it is prohibitively expensive to sample from this posterior. We produce approximate samples using a method described below.

b. Prior

We model C as a non-linear transformation of a Gaussian random field[8] $f(.)$, plus a random field $\varepsilon(.)$ that associates an independent normally distributed value with each location on the earth's surface. Specifically,

$$C(x) = g\big(f(x) + \epsilon(x)\big)$$

The link function *g* maps the random variable $f(x) + \epsilon(x)$, which can be any real number, to the interval (0,1), so $C(x)$ can be used as a probability or prevalence. We used a non-standard link function, which is described below.

The prior for *f* is parameterized so that the constant mean function $M(x) = m$, and the standard exponential covariance function $Cov(x,y) = \phi^2 \exp\frac{|x-y|}{\theta}$ with amplitude parameter φ and range parameter θ, with suitable priors assigned to the scalar parameters *m, φ* and *θ*:

$$p(m) \propto 1$$

$$\phi \sim Exponential(.1)$$

$$\theta \sim Exponential(.1)$$

$$f \sim GP(M, Cov)$$

The units of x, y and θ are earth radii, and m and φ are unitless. The unstructured component $\varepsilon(x)$ is modeled as normally distributed with unknown variance V:

$$V \sim Exponential(.1)$$

$$\epsilon(x) \overset{iid}{\sim} Normal(0, V, \alpha)$$

A regional approach was privileged over a global analysis due to the paucity of datapoints outside the African continent.

c. Likelihood

Adopting the Hardy-Weinberg assumption[3,4], if n_i individuals are sampled at the i'th observation location o_i (for a total of $2n_i$ chromosomes), the probability distribution for the number k_i of copies of the HbC allele that will be found is binomial, with probability $C(o_i)$:

$$k_i \sim Binomial(2n_i, C(o_i))$$

d. Flexible link function and empirical Bayesian analysis

The link function g for binomial data is usually taken to be the inverse logit function:

$$g(x) = logit^{-1}(x) = \frac{\exp x}{1 + \exp x}$$

Piel *et al.*[7] employed this model. Applying the change of variables formula, the induced prior for $C(x)$ is:

$$p\big(C(x)\big) = \frac{1}{C(x)\big(1 - C(x)\big)} Normal\big(logit\big(C(x)\big); m, \phi^2 + V\big)$$

Note that this is essentially a two-parameter family of probability distributions, since φ and V appear only in the sum.

When we initially attempted to fit Piel et al.'s model to the current dataset and predict CC, we found that, when the local distribution above is fitted in areas where datapoints are highly clustered, the best fitting values of m and $\phi^2 + V$ result in implausibly long right-hand tails for the predictive distribution of prevalence in the next observation at x. Although the standard summary statistics, including the upper 95% credible interval, were consistent with the local dataset, strikingly high allele frequencies $C(x)$ (greater than 30%) were predicted with small but practically significant probability (0.1% or so).

This particular type of lack of fit was not a major issue for predictions of $C(x)$ because the bulk of the predictive distribution was roughly consistent with the dataset, but the long right-hand tail translated to an even longer right-hand tail for $CC(x)$, which contained enough mass to skew all of the standard summary statistics. For example, the predicted mean of $CC(x)$ in some areas exceeded 5%, which is highest than all but the observed values in the dataset.

To remedy this problem, we attempted several strategies including employing Stukel's link function[9] in place of the more standard inverse logit, and modelling $f(x) + \epsilon(x)$ as a skew-Gaussian process[10] rather than the standard Gaussian. The skew-Gaussian approach showed indications that it would solve the problem, but eliminated the crucial conjugate relationship between $f(x)$ and $f(x) + \epsilon(x)$, and we were unable to devise a successful MCMC scheme.

Ultimately, we employed an alternative flexible link function:

$$h(x) = \sum_{i=0}^{3} \mathbb{C}_i x^i$$

$$g = logit^{-1} \circ h$$

We were unable to infer $\mathbb{C}_i$ jointly with the other model parameters in a fully Bayesian manner due to poor MCMC mixing, so we adopted an empirical fitting approach inspired by data pre-processing steps employed in classical geostatistics, which improved the fitting of the model to the

data. The polynomial coefficients for such function are specific to the dataset. The set of coefficients used, corresponding to an invertible function and fitting the empirical cumulative distribution function (CDF) was:

$$y = -0.072328175x^3 + 1.105591388x^2 + 0.048698858x + 0.004114882.$$

e. Empirical Bayesian approach to fitting the polynomial coefficients

For each observation, (n_i, k_i), we first obtained the posterior expectation of the gene pool-wide prevalence of HbC with uniform prior density on *[0,1]*:

$$\hat{p}_i = \frac{k_i + 1}{2n_i + k_i + 2}$$

We discarded values for which $2n_i$ was below 50. Then, we inferred the parameters $\tilde{m}$ and $\tilde{V}$ of the non-spatial Bayesian model:

$$p(\hat{p}) = \prod_i \frac{1}{\hat{p}_i(1-\hat{p}_i)} Normal\big(logit(\hat{p}_i); \tilde{m}, \tilde{V}\big)$$

We then plotted the posterior predictive CDF of $logit(\hat{p})$ against its empirical CDF, and fit the coefficients *Ȼ* of the cubic polynomial function *h* to the points using least squares, subject to the constraint that *h* must be invertible (or, equivalently, monotone).

In the Bayesian analysis of the full spatial model, the fitted values of Ȼ were taken as known and fixed. Although this empirical procedure is admittedly informal, the resulting nonstandard link function did substantially improve the fit of the model to the data.

f. Prior predictive constraint

Epidemiological data on haemoglobin C (HbC) tend to be opportunistic, i.e. relatively abundant in areas where it is expected to be found (i.e. West Africa), but rare elsewhere, even within Africa. Finding a carrier in a population survey conducted areas anywhere on this continent is nevertheless plausible.

Even with the more flexible model for *ε(x)*, the predictive distribution in areas of low data coverage exhibits long right-hand tails, and the predicted mean value of *C(x)* in these regions is surprisingly high. The ideal solution to this problem would be to gather further data on the prevalence of HbC in areas where data are missing from sources such as health service reports, and incorporate it in the model. Given the obvious logistical difficulties associated with this ideal approach and the lack of spatial precision when such data is available, we found it more practical to supplement the dataset with expert opinion.

Perhaps the best way to incorporate this expert opinion would be as 'soft data', as described by Christakos[11] among others. However, producing defensible local pseudo-observations of HbC allele frequency all but requires the data collection process that we sought to avoid. In addition, using soft datapoints would increase the number of spatial locations at which the Gaussian random field *f* has to be imputed, increasing the computational expense of fitting the model.

As a compromise, we elected to constrain *φ*, *m*, and *V* in such a way that the prior predictive distribution of *C(x)*, before the data are incorporated, puts probability mass of 1×10^{-4} or less on values in excess of .0001. In other words, we constrained 99.99% of the prior predictive probability mass between allele frequencies of 0% and 0.01%.This constraint arguably induces a lack of fit by forcing *f(x)* to depart from its prior mean by many standard deviations in areas where HbC allele frequency is known to be high; but it does remedy the implausibly high predictive values in some parts of Africa, and does not seem to adversely affect the fit in other areas.

Multiple combinations of the threshold allele frequency and maximum probability values were tested in order to assess the performance of the model (not shown). The parameters presented here

represented to best compromise in terms of i) lowering the prediction in peripheral areas for which no data was available; ii) visually checking the appearance of summary maps; iii) checking our areal estimates against existing estimates. This can be seen as an informal way of bringing national reporting data into the model without incorporating it directly; iv) checking the mean error and mean absolute error.

g. Fitting the model

The model was fitted using a Markov chain Monte Carlo algorithm[12] implemented in the programming language Python using the Bayesian analysis package PyMC[13].

The scalar parameters φ, m, θ and V were updated jointly using Haario, Saksman and Tamminen's adaptive Metropolis algorithm[14], as implemented by PyMC's AdaptiveMetropolis step method. Each value $\varepsilon(o_i)$ at observation location o_i was updated separately using the standard one-at-a-time Metropolis algorithm. The distribution of the Gaussian random field at the observation locations, $\{f(o_i)\}$, is conjugate to the distribution of $\{\epsilon(o_i) + f(o_i)\}$, so we updated $\{f(o_i)\}$ by sampling from its full conditional distribution. MCMC output parameter values are summarised in Supplementary Table S2.

h. Mapping procedure

Interpolating spatially sparse survey data to predict an allele frequency across a wide region results in predictions of which the level of certainty (or uncertainty) varies spatially as a function of the density, quality, and sample size of survey data available. Spatial heterogeneity of the frequency, known to be high in some areas for other haemoglobinopathies such as HbS[7,15], also influences this uncertainty. A Bayesian MBG framework[16] generates a posterior predictive distribution rather than a unique value, therefore allowing estimation of the uncertainty of the prediction for each pixel. In addition to the posterior predictive distributions of HbC allele frequency, HbAC and HbCC genotype frequencies were also generated directly by the model. Because these are non-linear functions of the allele frequency, it would be incorrect to produce summary maps of these quantities from those of allele frequency using GIS software[8].

The uncertainty is a crucial measure of the accuracy of the prediction. From the complete range of possible uncertainty intervals available from the model's output, we chose here to use the inter-quartile range (IQR) of the posterior distribution[17], corresponding to a 50% probability. This corresponds to the *mbg-map* command of the generic MBG package.

i. Mean vs. median

The output of the model is a full posterior predictive distribution (PPD). A multitude of summary statistics is therefore available.[8] The most common ones are the mean, the mode and the median.

The main advantage of the mean is that it can be used correctly to predict means of other quantities using GIS software, because the mean of a sum is equal to the sum of the means. The mean could therefore be used to compare the regional areal prediction with the sum of the national areal predictions. Because the estimates at national and regional scales were calculated independently, we did not expect to obtain equal values, but we expected them to be consistent with the Monte Carlo standard error (SE) obtained from the ten repetitions conducted at each scale. We therefore used the mean to check the sanity of our independent estimates at national and regional scales. All the sums of the mean areal estimates corresponding to sub-spatial units fell within the SE range of spatial units areal estimates.

The main advantage of the median is that it can be used in combination with the interquartile range to give a better picture of the overall prediction, particularly when the PPD is highly skewed, and its associated uncertainty. Because the sum of the median is not equivalent to the median of the sums, important differences can be observed between the regional estimate (AFRO) and the sum of national estimates. Although counter-intuitive, this is statistically correct in the present context.

j. Model validation

In addition to the model-based representations of prediction uncertainty provided by the MBG framework, the model's predictive ability was quantified by assessing the disparity between the prediction and the observed allele frequency using a validation subset of the data. Ten percent of the data (n=20), randomly selected, were held out from the dataset. The model was run in full using the thinned data set (n=186) to generate HbC PPD for comparison with known values at the locations of the held-out data (Supplementary Figure S3a). The prediction's mean error and mean absolute error were used to assess the model's overall bias and overall accuracy respectively. The mean error is the average distance between the actual data points and the predicted values. The absolute mean error is a measure of the average magnitude of the errors in the predicted values. A procedure was also implemented to test the extent to which predicted posterior distributions at each location provided a suitable measure of uncertainty. Working through 100 progressively narrower credible intervals (CIs), from the 99% CI to the 1% CI, each was tested by computing the actual proportion of held-out prevalence observations that fell within the predicted CI. In a perfect model, 95% of true values should fall within the 95% CI predicted at each location, 50% within the 50% CI, and so on. Plotting these actual proportions against each predicted CI level allows the overall fidelity of the posterior probability distributions predicted at the held-out data locations to be assessed (Supplementary Figure S3b). This corresponds to the *mbg-validate* command of the generic MBG package.

k. Demographic data

Population density is highly variable between pixels within one country. National estimates of HbC newborns therefore depend on whether areas of high or low frequencies are highly populated or not. Rather than using crudely averaged data for each country, the use of our contemporary allele frequency map for Africa combined with high resolution population data allows us to deal with this issue. Population density data have been described in detail in Balk *et al*.[18] and calculations to adjust them to 2010 populations explained in Gething *et al*.[19]

We focus here on newborns using Hardy-Weinberg assumptions[3,4]. Assuming random mating and large population sizes, it is possible to estimate the HbC allele frequency and the proportions of each genotype (AA, AC and CC) from the number of heterozygote individuals observed in the population sample[20]. Conceptually, the number of AC and CC babies born per year can be obtained by multiplying a function of HbC allele frequency ($2p(1-p)$ and p^2 respectively) by the population living within the area of interest and the crude birth rate (CBR). Crude birth rates are not consistently available across Africa at a finer resolution than the country level, hence the use of data from the United Nations Population Prospects for the 2010-2015 period[21].

l. Areal predictions

As described previously[8], one needs to be careful when predicting integrals over spatial areas. Using traditional GIS methods, a researcher having access to a map of HbC allele frequency ($C(x)$) and desiring a national proportion of individuals with the CC genotype ($CC(x)$), would take the square of $C(x)$ and then average the values over the various pixels falling within the country of interest, weighted by population. This approach has limitations when the map of allele frequency is uncertain. Squaring the mean map for $C(x)$ does not yield the mean map for $CC(x)$ and it is impossible to produce any assessment of the uncertainty of the areal average from summary maps alone[8].

To develop a correct procedure for producing predictive distributions for national proportions, we begin by considering what we would do if we had the true map of HbC allele frequency in hand. As stated above, the national-level disease burden $D(A)$ in nation A can be computed from C, the birth rate bA in A and the population density surface N using the areal integral:

$$D(A) = \int_A b_A N(x) C(x)^2 da \qquad (1)$$

In reality, the allele frequency map C is unknown. We do not know its exact value, but we have a posterior distribution $[C]$ for it, from which samples can be drawn. Because applying $equation\ 1$ to a sample from $[C]$ generates a sample from $[D(A)]$, many samples from $[C]$ can be used to build up

a histogram approximating $[D(A)]$. Summaries such as the mean, median, variance and credible intervals can be approximated using these samples.

Although it is mathematically correct, this procedure is impractical to implement. We have an approximation of $[C]$ in the form of the MCMC trace, but generating samples from it at an appropriately high resolution is extremely computationally expensive[8,22]. We use an approximate procedure based on the fact that, if z_1 is a single-element binomial process on A with intensity d,

$$\frac{\int_A C(x)^2 d(x) da}{\int_A d(x) da} = E(C(z_1)^2) \qquad (2)$$

Furthermore, if z_i is an l-element binomial process on a with intensity d,

$$E(C(z_1)^2) = \lim_{l \to \infty} \frac{1}{l} \sum_{i=1}^{l} g(C(z_i)^2) \qquad (3)$$

The expectation of the term inside the limit is equal to the left-hand term, but its variance is smaller than that of $C(z_1)$.

The pseudocode for our procedure, based on this approximation, was as follows:

1. Generate an l-element binomial process on A, z_l, with intensity N.
2. For each value in the thinned MCMC trace for the scalar parameters and the Gaussian random field f evaluated at the observation locations $\{o_i\}$, $\{f(o_i)\}$,
   - Draw a value for the *l*-element random vector $\{f(z_i)\}$ from its full conditional distribution.
   - Convert these values to $\{C(z_i)\}$ by applying the inverse-logit link function.
   - Square these values to obtain a sample for the value of the desired genotype frequencies $\{C(z_i)^2\}$.
   - Compute the arithmetic mean of this sample and store.

This procedure was conducted ten times using l = 5,000 and 1,000 spatial points for the regional and national areal estimates respectively; 10% of the parameter samples in the dynamic trace, selected at random; and 1,000 iterations. As this resulted in a full PPD for each areal unit of interest, various parameters could be used to summarize the predicted estimates and their uncertainty. Here, we used the median and the interquartile range (IQR).

The code used to implement this analysis is freely available at http://github.com/malaria-atlas project/ibd-world and http://github.com/malaria-atlas-project/generic-mbg.

m. Monte Carlo standard errors

To estimate the Monte Carlo standard error[12,16,23] attributable to the use of a set of l spatial locations rather than a high-resolution raster grid, we repeated all computations ten times and recorded the sample standard deviations of all summaries (mean, median, etc.). The point estimates that we report were obtained by aggregating the samples from all repetitions. The Monte Carlo standard errors for the national and regional areal estimates are summarised in Supplementary Table S1.

References

1 Bain, B. J. *Haemoglobinopathy Diagnosis*. Second edn, (Blackwell Publishing Ltd, 2006).

2 Hay, S. I. *et al.* A world malaria map: *Plasmodium falciparum* endemicity in 2007. *Public Library of Science Medecine* **6**, e1000048 (2009).

3 Hardy, G. H. Mendelian proportions in a mixed population. *Science* **28**, 49-50 (1908).

4 Weinberg, W. Über den Nachweis der Vererbung beim Menschen. *Jahreshefte des Vereins für Vaterländische Naturkunde in Württemberg* **64**, 369-382 (1908).

5 Bittles, A. H. Consanguinity and its relevance to clinical genetics. *Clinical Genetics* **60**, 89-98, doi:10.1034/j.1399-0004.2001.600201.x (2001).

6 Guerra, C. *et al.* Assembling a global database of malaria parasite prevalence for the Malaria Atlas Project. *Malaria journal* **6**, 17 (2007).

7 Piel, F. B. *et al.* Global distribution of the sickle cell gene and geographical confirmation of the malaria hypothesis. *Nature Communications* **1**, 104 (2010).

8 Patil, A. P., Gething, P. W., Piel, F. B. & Hay, S. I. Bayesian geostatistics in health cartography: the perspective of malaria. *Trends in Parasitology* **27**, 246-253 (2011).

9 Stukel, T. A. Generalized Logistic Models. *Journal of the American Statistical Association* **83**, 426-431 (1988).

10 Azzalini, A. & Capitanio, A. Statistical applications of the multivariate skew normal distribution. *Journal of the Royal Statistical Society: Series B (Statistical Methodology)* **61**, 579-602, doi:10.1111/1467-9868.00194 (1999).

11 Christakos, G. *Modern Spatiotemporal Geostatistics*. 217 (Oxford University Press, 2000).

12 Gilks, W. R., Richardson, S. & Spiegelhalter, D. *Markov chain Monte Carlo in Practice*. (Chapman & Hall/CRC, 1995).

13 Patil, A. P., Huard, D. & Fonnesbeck, C. J. PyMC: Bayesian stochastic modelling in Python. *Journal of Statistical Software* **35**, 1-81 (2010).

14 Haario, H., Saksman, E. & Tamminen, J. An Adaptive Metropolis Algorithm. *Bernoulli* **7**, 223-242 (2001).

15 Livingstone, F. B. *Frequencies of Hemoglobin Variants: Thalassemia, the Glucose-6-Phosphate Dehydrogenase Deficiency, G6Pd Variants and Ovalocytosis in Human Populations*. (Oxford University Press, 1985).

16 Diggle, P. J. & Ribeiro Jr, P. J. *Model-based geostatistics*. (Springer, 2007).

17 Patil, A. P. *et al.* Defining the relationship between *Plasmodium falciparum* parasite rate and clinical disease: statistical models for disease burden estimation. *Malaria journal* **8**, 186, doi:10.1186/1475-2875-8-186 (2009).

18 Balk, D. L. *et al.* Determining global population distribution: methods, applications and data. *Advances in Parasitology* **62**, 119-156 (2006).

19 Gething, P. W. *et al.* A new world malaria map: *Plasmodium falciparum* endemicity in 2010. *Malaria journal* **10**, 378 (2011).

20 Grosse, S. D. *et al.* Sickle cell disease in Africa: a neglected cause of early child mortality. *American Journal of Preventive Medicine* **41**, S398-405 (2011).

21 United Nations world population prospects: The 2010 Revision. . http://esa.un.org/unpp (Accessed: 25 July 2011)

22 Gething, P. W., Patil, A. P. & Hay, S. I. Quantifying aggregated uncertainty in *Plasmodium falciparum* malaria prevalence and populations at risk *via* efficient space-time geostatistical joint simulation. *Public Library of Science Computational Biology* **6**, e1000724 (2010).

23 Moran, P. A. P. The estimation of standard errors in Monte Carlo simulation experiments. *Biometrika* **62**, 1-4, doi:10.1093/biomet/62.1.1 (1975).

Supplementary References

List of the 174 references from which HbC data have been used as input into our map of data points. References are ordered alphabetically by surname.

1 Acemoglu, H., Beyhun, N. E., Vancelik, S., Polat, H. & Guraksin, A. Thalassaemia screening in a non-prevalent region of a prevalent country (Turkey): is it necessary? *Public Health* **122**, 620-624, doi:10.1016/j.puhe.2007.09.007 (2008).

2 Acquaye, C. T. A. & Oldham, J. H. Variants of haemoglobin and glucose-6-phosphate dehydrogenase - I. Distribution in Southern Ghana. *Ghana Medical Journal* **12**, 412-418 (1973).

3 Adeyemo, O. A. & Soboyejo, O. B. Frequency distribution of ABO, RH blood groups and blood genotypes among the cell biology and genetics students of University of Lagos, Nigeria. *African Journal of Biotechnology* **5**, 2062-2065 (2006).

4 Ágreda, F. *et al.* Frecuencia de Portadores del Alelo S en la Población de 15 - 49 Años de Portobelo. Panamá. Junio 2004. *Revista Médico Científica* **17**, 66-70 (2004).

5 Ahern, E. J., Swan, A. V. & Ahern, V. N. The prevalence of the rarer inherited haemoglobin defects in adult Jamaicans. *British Journal of Haematology* **25**, 437-444 (1973).

6 Akinkugbe, F. M. Anaemia in a rural population in Nigeria (Ilora). *Annals of Tropical Medicine and Parasitology* **74**, 625-633 (1980).

7 Alkindi, S. *et al.* Forecasting hemoglobinopathy burden through neonatal screening in Omani neonates. *Hemoglobin* **34**, 135-144, doi:10.3109/03630261003677213 (2010).

8 Allen, S. J. *et al.* Morbidity from malaria and immune responses to defined *Plasmodium falciparum* antigens in children with sickle cell trait in The Gambia. *Trans R Soc Trop Med Hyg* **86**, 494-498 (1992).

9 Allison, A. C. The sickle-cell and haemoglobin C genes in some African populations. *Annals of Human Genetics* **21**, 67-89 (1956).

10 Almeida, A. M., Henthorn, J. S. & Davies, S. C. Neonatal screening for haemoglobinopathies: the results of a 10-year programme in an English Health Region. *British Journal of Haematology* **112**, 32-35 (2001).

11 Al-Nood, H., Al-Ismail, S., King, L. & May, A. Prevalence of the sickle cell gene in Yemen: a pilot study. *Hemoglobin* **28**, 305-315 (2004).

12 al-Nuaim, L., Talib, Z. A., el-Hazmi, M. A. & Warsy, A. S. Sickle cell and G-6-PD deficiency gene in cord blood samples: experience at King Khalid University Hospital, Riyadh. *Journal of tropical pediatrics* **43**, 71-74 (1997).

13 Arends, A. Data for Venezuela. *Personal communication* (2010).

14 Arends, T. Absence of abnormal haemoglobins in Colombian Tunebo Indians. *Nature* **190**, 93-94 (1961).

15 Arends, T. Estado actual del estudio de las hemoglobinas anormaltes en Venezuela. *Sangre* **8**, 1-14 (1963).

16 Arends, T. Frecuencia de hemoglobinas anormales en poblacionas humanas sudamericanas. *Acta Cientifica Venezolana* **14**, 46-57 (1963).

17 Arends, T. *et al.* Intratribal genetic differentiation among the Yanomama Indians of southern Venezuela. *Proceedings of the National Academy of Sciences of the United States of America* **57**, 1252-1259 (1967).

18 Arends, T., Gallango, M. L., Muller, A., Gonzalez-Marroro, M. & Perez Bandez, O. in *International Congress on Anthropological and Ethnological Sciences.* 1.

19 Aung Than, B. & Pe, U. H. Haemoglobinopathies in Burma. I. The incidence of haemoglobin E. *Tropical and Geographical Medicine* **23**, 15-19 (1971).

20 Auricchio, M. T. B. D. M., Vicente, J. P., Meyer, D. & Mingroni-Netto, R. C. Frequency and origins of hemoglobin S mutation in African-derived Brazilian populations. *Human Biology* **79**, 667-677 (2007).

21 Azevedo, E. S. *et al.* Distribution of abnormal hemoglobins and glucose-6-phosphate dehydrogenase variants in 1200 school children of Bahia, Brazil. *American Journal of Physical Anthropology* **53**, 509-512 (1980).

22 Azevedo, E. S. *et al.* Genetic and anthropological studies in the island of Itaparica, Bahia, Brazil. *Human Heredity* **31**, 353-357 (1981).

23 Baghernajad-Salehi, L. *et al.* A pilot beta-thalassaemia screening program in the Albanian population for a health planning program. *Acta Haematologica* **121**, 234-238, doi:10.1159/000226423 (2009).

24 Balgir, R. S. The spectrum of haemoglobin variants in two scheduled tribes of Sundargarh district in north-western Orissa, India. *Ann Hum Biol* **32**, 560-573, doi:10.1080/03014460500228741 (2005).

25 Ballas, S. K., Park, D. & Wapner, R. J. Neonatal screening for sickle cell disease in a metropolitan university hospital: efficacy and problems. *Journal of medical screening* **1**, 229-232 (1994).

26 Barnicot, N. A. *et al.* Haemoglobin types in Greek populations. *Annals of Human Genetics* **26**, 229-236 (1963).

27 Basu, A. *et al.* Morphology, serology, dermatoglyphics, and microevolution of some village populations in Haiti, West Indies. *Human Biology* **48**, 245-269 (1976).

28 Basu, S., Kumar, A., Sachdeva, M. P. & Saraswathy, K. N. Incidence of NESTROFT-positives and haemoglobin S among the Jats and Brahmins of Sampla, Haryana. *Anthropologist* **10**, 203-205 (2008).

29 Beall, C. M. *et al.* An Ethiopian pattern of human adaptation to high-altitude hypoxia. *Proc Natl Acad Sci U S A* **99**, 17215-17218, doi:10.1073/pnas.252649199 (2002).

30 Bernstein, S. C., Bowman, J. E. & Kaptue Noche, L. Population studies in Cameroon: hbs, G-6pd deficiency and falciparum malaria. *Human Heredity* **30**, 251-258 (1980).

31 Beuzard, Y. *et al.* Dépistage des hémoglobinopathies dang le sang de cordon par isoélectrofocalisation. *Nouvelle Revue Française d'Hématologie* **21**, 126 (1979).

32 Bienzle, U. *et al.* The distribution and interaction of haemoglobin variants and the beta thalassaemia gene in Liberia. *Human Genetics* **63**, 400-403 (1983).

33 Bienzle, U., Okoye, V. C. & Gogler, H. Haemoglobin and glucose-6-phosphate dehydrogenase variants: distribution in relation to malaria endemicity in a Togolese population. *Z Tropenmed Parasitol* **23**, 56-62 (1972).

34 Biondi, G. *et al.* Distribution of the S and C hemoglobins in Atakora District (Benin). *Hum Biol* **52**, 205-213 (1980).

35 Blackwell, R. Q., Tung-Hsiang Huang, J. & Chien, L. C. Abnormal hemoglobin characteristics of Taiwan aborigines. *Hum Biol* **37**, 343-356 (1965).

36 Boemer, F., Vanbellinghen, J. F., Bours, V. & Schoos, R. Screening for sickle cell disease on dried blood: a new approach evaluated on 27,000 Belgian newborns. *Journal of medical screening* **13**, 132-136 (2006).

37 Bouyou-Akotet, M. K. *et al.* Prevalence of *Plasmodium falciparum* infection in pregnant women in Gabon. *Malaria journal* **2**, 18 (2003).

38 Boyer, S. H., Crosby, E. F., Fuller, G. F., Ulenurm, L. & Buck, A. A. A survey of hemoglobins in the Republic of Chad and characterization of hemoglobin Chad:alpha-2-23Glu--Lys-beta-2. *Am J Hum Genet* **20**, 570-578 (1968).

39 Boyo, A. E. Starch gel electrophoresis of the haemoglobin of Nigerian children. A preliminary investigation of the incidence of thalassaemia. *The West African medical journal* **12**, 75-81 (1963).

40 Bruce-Tagoe, A. A. *et al.* Haematological values in a rural Ghanaian population. *Tropical and Geographical Medicine* **29**, 237-244 (1977).

41 Buckle, A. E. & Holman, C. A. Routine Haemoglobin Electrophoresis in at-Risk Gravid Women. *J Obstet Gynaecol Br Commonw* **71**, 923-926 (1964).

42 Buettner Janusch, J., Bove, J. R. & Young, N. Genetic Traits and Problems of Biological Parenthood in Two Peruvian Indian Tribes. *American Journal of Physical Anthropology* **22**, 149-154 (1964).

43 Buettner-Janusch, J. & Buettner-Janusch, V. Hemoglobins, haptoglobins, and transferrins in indigenous populations of Kenya. *American Journal of Physical Anthropology* **32**, 27-32 (1970).

44 Cabannes, R. in *Abnormal Haemoglobins in Africa* (ed J. H. P. Jonxis) 291-317 (Davis, F. A., 1965).

45 Cabannes, R., Beurrier, A. & Larrouy, G. La thalassémie chez les indiens de Guyane Française. *Nouvelle Revue Française d'Hématologie* **5**, 617-629 (1965).

46 Cabannes, R., Larrouy, G., Fernet, P. & Sendrail, A. Etude hémotypologique des populations sédentaires de la Saoura (Sahara occidental). II. Les hémoglobines. *Bulletins et Mémoires de la Société d'Anthropologie de Paris* **12**, 139-142 (1969).

47 Cabannes, R., Larrouy, G. & Sendrail, A. Etude hémotypologique des populations du massif du Hoggar et du plateau de l'Air. III. Les hémoglobines. *Bulletins et Mémoires de la Société d'Anthropologie de Paris* **12**, 143-146 (1969).

48 Cabannes, R., Lefevre-Witier, P. & Sendrail, A. III. Etude des hémoglobines dans les populations du Tassili N'Ajjer. *Bulletins et Mémoires de la Société d'Anthropologie de Paris* **12**, 434-439 (1967).

49 Cabannes, R., Nicolas, C., Houvet, D., Fellenz, C. & Sangare, A. Etude hémotypologique et biologique des Abrons et des métis Koulango-abron. *Annales de l'Université d'Abidjan - Faculté de Médecine* **12**, 119-141 (1978).

50 Cabannes, R. & Schmidt-Beurrier, A. Recherches sur les haemoglobines des populations indiennes de l'Amerique de Sud. *L'Anthropologie* **70**, 331-334 (1966).

51 Cabannes, R., Schmidt-Beurrier, A. & Monnet, B. Etude des protéines, des haptoglobines, des transferrines et des hémoglobines d'une population noire de Guyane française (Boni). *Bulletin de la Société de Pathologie Exotique et de ses Filiales* **59**, 908-916 (1966).

52 Cabannes, R. *et al.* Etude hémotypologique et biologique des Attié du village d'Atiekwa. *Médecine d'Afrique Noire* **17**, 835-841 (1970).

53 Cabannes, R., Sy, B. & Schmidt-Beurrier, A. Etude des hémoglobinoses dans la région de Niamey (Moyen Niger). *Nouvelle Revue Française d'Hématologie* **7**, 309-313 (1967).

54 Calvo-Villas, J. M. *et al.* Prevalencia de hemoglobinopatias en mujeres gestantes en el area sanitaria de Lanzarote. *Anales de Medicina Interna* **23**, 206-212 (2006).

55 Cao, A. *et al.* Thalassaemia and glucose-6-phosphate dehydrogenase screening in 13- to 14-year-old students of the Sardinian population: preliminary findings. *Community genetics* **11**, 121-128, doi:10.1159/000113873 (2008).

56 Chasen, S. T., Loeb-Zeitlin, S. & Landsberger, E. J. Hemoglobinopathy screening in pregnancy: comparison of two protocols. *American journal of perinatology* **16**, 175-180 (1999).

57 Chaudhuri, S. *et al.* in *Proceedings of the 9th Congress of the International Society of Blood Transfusion.* (ed Totopara) 196-205 (Karger, S.).

58 Chopra, J. G. & Byam, N. T. Anemia survey in Trinidad and Tobago. *Am J Public Health Nations Health* **58**, 1922-1936 (1968).

59 Compri, M. B., Polimeno, N. C., Stella, M. B. & Ramalho, A. S. Programa comunitário de hemoglobinopatias hereditárias em população estudantil brasileira. *Revista de Saude Publica* **30**, 187-195 (1996).

60 Cresta, M. & Avoundogba, N. Risultati dello studio longitdinale dalla nasciia a 5 anni in un gruppo di bambini di Porto Novo (Repubblica Popolare del Benin). *Rivista di Antropologia* **61**, 43-132 (1980).

61 Cresta, M., Spedini, G. & Dlivieri, V. Antropologia morfologica ed emalogica del basso Dahomey. Nota III- Emazie. emoglobine, caratteri chimici. *Rivista di Antropologia* **55**, 189-202 (1968).

62 Crompton, P. D. *et al.* Sickle cell trait is associated with a delayed onset of malaria: implications for time-to-event analysis in clinical studies of malaria. *The Journal of infectious diseases* **198**, 1265-1275, doi:10.1086/592224 (2008).

63 Daudt, L. E. *et al.* Triagem neonatal para hemoglobinopatias: um estudo piloto em Porto Alegre, Rio Grande do Sul, Brasil. *Cadernos de saude publica / Ministerio da Saude, Fundacao Oswaldo Cruz, Escola Nacional de Saude Publica* **18**, 833-841 (2002).

64 de Araujo, M. C., Serafim, E. S., de Castro Jr, W. A. & de Medeiros, T. M. Prevalencia de hemoglobinas anormais em recem-nascidos da cidade de Natal, Rio Grande do Norte, Brasil. *Cadernos de saude publica / Ministerio da Saude, Fundacao Oswaldo Cruz, Escola Nacional de Saude Publica* **20**, 123-128 (2004).

65 De Bernal, M., Collazos, A., Bonilla, R. D. & Tascon, E. P. Determination of the prevalence of hemoglobin S, C, D, and G in neonates from Buenaventura, Colombia. *Colomb Medica* **41**, 141-147 (2010).

66 de Pinango, C. L. A. & Arends, T. Abnormal haemoglobins in native blood donors of Bolivar State. *Acta Cientifica Venezolana* **16**, 215-218 (1965).

67 de Souza, R. A. V., Pratesi, R. & Fonseca, S. F. Programa de triagem neonatal para hemoglobinopatias em Dourados, MS – uma análise. *Revista Brasileira de Hematologia e Hemoterapia* **32**, 126-130, doi:10.1590/s1516-84842010005000037 (2010).

68 Devoucoux, R. *et al.* Population genetics of abnormal haemoglobins in Burkina Faso, west Africa. *Ann Hum Biol* **18**, 295-302 (1991).

69 Deyde, V. M. *et al.* Epidemiological profile of hemoglobinopathies in the Mauritanian population. *Ann Hematol* **81**, 320-321 (2002).

70 Dufrenot & Legait, J. P. Contribution à l'étude de la répartition des genes S et C hémoglobiniques en Haute-Volta, au Mali et au Niger. *Bulletin de la Société de Pathologie Exotique et de ses Filiales* **63**, 606-614 (1970).

71 Edington, G. M. in *C.I.O.M.S. Symposium on Abnormal Haemogllobins* (eds J.H.P. Jonxis & J.F. Delafresnaye) 290-299 (Blackwell Scientific Publications, 1959).

72 Edington, G. M. & Laing, W. N. Relationship between haemoglobins C and S and malaria in Ghana. *Br Med J* **2**, 143-145 (1957).

73 Enevold, A. *et al.* Associations between alpha+-thalassemia and *Plasmodium falciparum* malarial infection in northeastern Tanzania. *The Journal of infectious diseases* **196**, 451-459 (2007).

74 Etcheverry, R. *et al.* Investigacion de grupos sanguineos y otros caracteres geneticos sanguineos en indigenas de Chile. I. En Atacamenos y Mapuches. *Rev Med Chil* **95**, 599-604 (1967).

75 Evans, D. I. & Blair, V. M. Neonatal screening for haemoglobinopathy. Results in 7691 Manchester newborns. *Archives of Disease in Childhood* **51**, 127-130 (1976).

76 Ewing, N., Powars, D., Hilburn, J. & Schroeder, W. A. Newborn diagnosis of abnormal hemoglobins from a large municipal hospital in Los Angeles. *Am J Public Health* **71**, 629-631 (1981).

77 Ezeilo, G. C. Sickle-cell trait frequency in Zambia. *Tropical and Geographical Medicine* **22**, 189-197 (1970).

78 Fabritius, H., Millan, J. & Le Corroller, Y. Dépistage systématique des hémoglobinopathies chez les donneurs de sang de la Guadeloupe (Antilles Françaises). *Rev Fr Transfus Immunohematol* **21**, 937-950 (1978).

79 Facer, C. A. & Brown, J. Incidence of abnormal haemoglobin traits among Gambian children. *Trans R Soc Trop Med Hyg* **73**, 309-311 (1979).

80 Farzana, F., Zuberi, S. J. & Hashmi, J. A. Prevalence of abnormal hemoglobin and thalassemia trait in a group of professional blood donors and hospital staff in Karachi. *J Pak Med Assoc* **25**, 237-239 (1975).

81 Fattoum, S. Les hémoglobinopathies en Tunisie. Revue actualisée des données épidémiologiques et moléculaires. *La Tunisie medicale* **84**, 687-696 (2006).

82 Firschein, I. L. Population dynamics of the sickle-cell trait in the Black Caribs of British Honduras, Central America. *Am J Hum Genet* **13**, 233-254 (1961).

83 Fleming, A. F., Storey, J., Molineaux, L., Iroko, E. A. & Attai, E. D. Abnormal haemoglobins in the Sudan savanna of Nigeria. I. Prevalence of haemoglobins and relationships between sickle cell trait, malaria and survival. *Annals of Tropical Medicine and Parasitology* **73**, 161-172 (1979).

84 Foster, K., Forbes, M., Hayes, R. & Serjeant, G. R. Cord blood screening for sickle hemoglobin: evidence against a female preponderance of hemoglobin S. *Journal of Pediatrics* **98**, 79-81 (1981).

85 Gamet, A. Première étude sur les hémoglobinoses au centre-Cameroun. *Bulletin de la Société de Pathologie Exotique et de ses Filiales* **57**, 1125-1133 (1964).

86 Gatti, F., Van Ros, G. & Vandepitte, J. Hémoglobines anormales à la maternité de Kinshasa. Une nouvelle famille congolaise avec hémoglobine. *Annales de la Societe Belge de Medecine Tropicale* **50**, 595-612 (1970).

87 Gentilini, M., M'Bengue, J. L., Danis, M. & Richard-Lenoble, D. Résultats de l'étude de l'électrophorèse de l'hémoglobine chez 500 camerounais de la région de Foumbot (Cameroun Oriental). *Med Trop (Mars)* **32**, 579-585 (1972).

88 Gouagna, L. C. *et al.* Genetic variation in human HBB is associated with *Plasmodium falciparum* transmission. *Nature genetics* **42**, 328-331, doi:10.1038/ng.554 (2010).

89 Gulbis, B. *et al.* Neonatal haemoglobinopathy screening in Belgium. *J Clin Pathol* **62**, 49-52, doi:10.1136/jcp.2008.060517 (2009).

90 Haj Khelil, A. *et al.* Clinical and molecular aspects of haemoglobinopathies in Tunisia. *Clinica chimica acta; international journal of clinical chemistry* **340**, 127-137 (2004).

91 Halberstein, R. A., Davies, J. E. & Mack, A. K. Hemoglobin variations on a small Bahamian island. *American Journal of Physical Anthropology* **55**, 217-221 (1981).

92 Hassan, M. K., Taha, J. Y., Al-Naama, L. M., Widad, N. M. & Jasim, S. N. Frequency of haemoglobinopathies and glucose-6-phosphate dehydrogenase deficiency in Basra. *Eastern Mediterranean health journal = La revue de sante de la Mediterranee orientale = al-Majallah al-sihhiyah li-sharq al-mutawassit* **9**, 45-54 (2003).

93 Henthorn, J., Anionwu, E. & Brozovic, M. Screening cord blood for sickle haemoglobinopathies in Brent. *Br Med J (Clin Res Ed)* **289**, 479-480 (1984).

94 Ibrahim, W. N. *et al.* Hereditary blood factors and anthropometry of the inhabitants of the Egyptian Siwa Oasis. *Hum Biol* **46**, 57-68 (1974).

95 Jain, R. C. Haemoglobinopathies in Libya. *J Trop Med Hyg* **82**, 128-132 (1979).

96 Jara, N. O., Guevara Espinoza, A. & Guderian, R. H. Investigacion de hemoglobinas anormales en poblaciones ecuatorianas de raza negra. *Sangre* **34**, 10-13 (1989).

97 Jeremiah, Z. A. Abnormal haemoglobin variants, ABO and Rh blood groups among student of African descent in Port Harcourt, Nigeria. *African health sciences* **6**, 177-181 (2006).

98 Joyanes, B. *et al.* Cribado de hemoglobinopatias en una cohorte de recien nacidos en la Comunidad de Madrid. *Medicina clinica* **126**, 290-292 (2006).

99 Junien, C. *et al.* Glucose-6-phosphate dehydrogenase and hemoglobin variants in Kel Kummer Tuareg and related groups. Indirect evidence for alpha-thalassemia trait. *Human Heredity* **32**, 318-328 (1982).

100 Kadkhodaei Elyaderani, M. *et al.* Ethnicity study and non-selective screening for haemoglobinopathies in the antenatal population of central Manchester. *Clinical and laboratory haematology* **20**, 207-211 (1998).

101 Kamel, K. *et al.* Anthropological studies among Libyans. Erythrocyte genetic factors, serum haptoglobin phenotypes and anthropometry. *American Journal of Physical Anthropology* **43**, 103-111 (1975).

102 Kaufman, M., Steier, W., Applewhaite, F., Ruggiero, S. & Ginsberg, V. Sickle-Cell Trait in Blood Donors. *The American journal of the medical sciences* **249**, 56-61 (1965).

103 Kirk, R. L., Lai, L. Y., Vos, G. H. & Vidyarthi, L. P. A genetical study of the Oraons of the Chota Nagpur Plateau (Bihar, India). *American Journal of Physical Anthropology* **20**, 375-385 (1962).

104 Kirk, R. L., Lai, L. Y., Vos, G. H., Wickremasinghe, R. L. & Perera, D. J. The blood and serum groups of selected populations in South India and Ceylon. *American Journal of Physical Anthropology* **20**, 485-479 (1962).

105 Kobbe, R. *et al.* A randomized controlled trial of extended intermittent preventive antimalarial treatment in infants. *Clin Infect Dis* **45**, 16-25, doi:10.1086/518575 (2007).

106 Kofi Ekue, J. M., Wurapa, F. K. & Rwabwogo-Atenyi, J. Haemoglobin genotypes, glucose - 6 - phosphate dehydrogenase deficiency and bronchial asthma. *East Afr Med J* **60**, 676-678 (1983).

107 Kulkarni, A. G. & Jekeme, S. D. Cord blood screening for haemoglobinopathies in northern Nigeria. *Annals of Tropical Medicine and Parasitology* **80**, 549-551 (1986).

108 Labie, D., Richin, C., Pagnier, J., Gentilini, M. & Nagel, R. L. Hemoglobins S and C in Upper Volta. *Human Genetics* **65**, 300-302 (1984).

109 Lallemant, M. *et al.* Hemoglobin abnormalities. An evaluation on new-born infants and their mothers in a maternity unit close to Brazzaville (P.R. Congo). *Human Genetics* **74**, 54-58 (1986).
110 Le Gallais, D. *et al.* Prevalence of the sickle cell trait among students in a physical education college in Côte-d'Ivoire. *Nouvelle Revue Française d'Hématologie* **31**, 409-412 (1989).
111 Lelong, M., Kaddari, F., Hanichi, A. & Porte, P. Intérêt du dépistage néonatal systmématique des hémoglobinopathies à l'Hôpital de Saint-Denis (93). *Revue Française des Laboratoires* **2001**, 19-21 (2001).
112 Lima, A. M. M. D., Azevedo, E., Krieger, H., Cabello, P. H. & Pollitzer, W. S. Admixture and relationships of the population of Jacobina, Bahia, Brazil. *American Journal of Human Biology* **8**, 483-488 (1996).
113 Lisker, R., Cordova, M. S. & Graciela Zarate, Q. B. Studies on several genetic hematological traits of the Mexican population. XVI. Hemoglobin, S and glucose-6-phosphate dehydrogenase deficiency in the east coast. *American Journal of Physical Anthropology* **30**, 349-354 (1969).
114 Lisker, R., Loria, A. & Cordova, M. S. Studies on Several Genetic Hematological Traits of the Mexican Population. 8. Hemoglobin S, Glucose-6-Phosphate Dehydrogenase Deficiency, and Other Characteristics in a Malarial Region. *Am J Hum Genet* **17**, 179-187 (1965).
115 Lorey, F. W., Arnopp, J. & Cunningham, G. C. Distribution of hemoglobinopathy variants by ethnicity in a multiethnic state. *Genetic epidemiology* **13**, 501-512 (1996).
116 Marsh, K., Otoo, L., Hayes, R. J., Carson, D. C. & Greenwood, B. M. Antibodies to blood stage antigens of *Plasmodium falciparum* in rural Gambians and their relation to protection against infection. *Trans R Soc Trop Med Hyg* **83**, 293-303 (1989).
117 Masmas, T. N. *et al.* Inherited hemoglobin disorders in Guinea-Bissau, West Africa: a population study. *Hemoglobin* **30**, 355-364 (2006).
118 Matson, G. A., Sutton, H. E., Swanson, J. & Robinson, A. Distribution of hereditary blood groups among Indians in South America. II. In Peru. *American Journal of Physical Anthropology* **24**, 325-349 (1966).
119 Mauran-Sendrail, A., Bouloux, C., Gomila, J. & Langaney, A. Comparative study of haemoglobin types of two populations of eastern Senegal--Bedik and Niokholonko. *Ann Hum Biol* **2**, 129-136 (1975).
120 MendonçaI, A. C., GarciaI, J. L., AlmeidaI, C. M., MegidI, T. B. C. & Fabron Júnior, A. Muito além do "Teste do Pezinho". *Revista Brasileira de Hematologia e Hemoterapia* **31**, 88-93 (2009).
121 Menendez, C. *et al.* The response to iron supplementation of pregnant women with the haemoglobin genotype AA or AS. *Trans R Soc Trop Med Hyg* **89**, 289-292 (1995).
122 Mercier, J., Romana, G. & Le Correller, Y. Prévalence de la drépanocytose en Guadeloupe. *Médecine Tropicale* **42**, 611-615 (1982).
123 Merghoub, T. *et al.* Haemoglobin D-Ouled Rabah among the Mozabites: a relevant variant to trace the origin of Berber-speaking populations. *European Journal of Human Genetics* **5**, 390-396 (1997).
124 Mockenhaupt, F. P. *et al.* Anaemia in pregnant Ghanaian women: importance of malaria, iron deficiency, and haemoglobinopathies. *Trans R Soc Trop Med Hyg* **94**, 477-483 (2000).
125 Monekosso, G. L. Clinical Survey of a Yoruba Village. *The West African medical journal* **13**, 47-59 (1964).
126 Monekosso, G. L. & Ibiama, A. A. Splenogamy and sickle-cell trait in a malaria-endemic village. *Lancet* **1** (1966).
127 Mutesa, L. *et al.* Neonatal screening for sickle cell disease in Central Africa: a study of 1825 newborns with a new enzyme-linked immunosorbent assay test. *Journal of medical screening* **14**, 113-116 (2007).
128 Neel, J. V. *et al.* Data on the occurrence of hemoglobin C and other abnormal hemoglobins in some African populations. *Am J Hum Genet* **8**, 138-150 (1956).
129 Nguematcha, R., Savina, J. F., Juhan, I., Boche, R. & Ravisse, P. Recherche de la tare drépanocytaire dans un groupe Pygmée du Sud Cameroun. *Médecine d'Afrique Noire* **20**, 605-606 (1973).
130 North, M. L., Piffaut, M. C., Duwig, I., Locoh-Donou, A. G. & Locoh-Donou, A. M. Detection of haemoglobinopathies at birth in Togo. *Nouvelle Revue Française d'Hématologie* **30**, 237-241 (1988).

131 Nurse, G. T., Jenkins, T., David, J. H. & Steinberg, A. G. The Njinga of Angola: a serogenetic study. *Ann Hum Biol* **6**, 337-348 (1979).
132 Odunvbun, M. E., Okolo, A. A. & Rahimy, C. M. Newborn screening for sickle cell disease in a Nigerian hospital. *Public Health* **122**, 1111-1116, doi:10.1016/j.puhe.2008.01.008 (2008).
133 Ogala, W. N. Haematological values in healthy Nigerian infants. *Annals of tropical paediatrics* **6**, 63-66 (1986).
134 Ohene-Frempong, K., Oduro, J., Tetteh, H. & Nkrumah, F. Screening newborns for sickle cell disease in Ghana. *Pediatrics* **121**, S120-S121 (2008).
135 Omotade, O. O. *et al.* Routine screening for sickle cell haemoglobinopathy by electrophoresis in an infant welfare clinic. *West African journal of medicine* **17**, 91-94 (1998).
136 Oomen, J. M., Meuwissen, J. H. & Gemert, W. Differences in blood status of three ethnic groups inhabiting the same locality in Northern Nigeria. Anaemia, splenomegaly and associated causes. *Tropical and Geographical Medicine* **31**, 587-606 (1979).
137 Parikh, S., Dorsey, G. & Rosenthal, P. J. Host polymorphisms and the incidence of malaria in Ugandan children. *The American journal of tropical medicine and hygiene* **71**, 750-753 (2004).
138 Piliszek, T. S. Hb Bart's and its significance in the South African Negro. *Acta Haematologica* **61**, 33-38 (1979).
139 Platt, O. S. *et al.* Mortality In Sickle Cell Disease -- Life Expectancy and Risk Factors for Early Death. *New England Journal of Medicine* **330**, 1639-1644, doi:doi:10.1056/NEJM199406093302303 (1994).
140 Pollitzer, W. S., Chernoff, A. I., Horton, L. L. & Froehlich, M. Hemoglobin patterns in American Indians. *Science* **129**, 216 (1959).
141 Pollitzer, W. S., Namboodiri, K. K., Elston, R. C., Brown, W. H. & Leyshon, W. C. The Seminole Indians of Oklahoma: morphology and serology. *American Journal of Physical Anthropology* **33**, 15-29 (1970).
142 Pollitzer, W. S. *et al.* The Seminole Indians of Florida: morphology and serology. *American Journal of Physical Anthropology* **32**, 65-81 (1970).
143 Quadri, M. I., Islam, S. I. & Nasserullah, Z. The effect of alpha-thalassemia on cord blood red cell indices and interaction with sickle cell gene. *Annals of Saudi medicine* **20**, 367-370 (2000).
144 Raccurt, C. P. *et al.* Prévalence des hémoglobines anormales en Haiti: Sondage au sein d'une population rurale de la Plaine du Cul-de-Sac. *Annales de la Societe Belge de Medecine Tropicale* **63**, 241-246 (1983).
145 Rahbar, S. & Blume, K. Hemoglobinopathies in the Los Angeles area. *Hemoglobin* **7**, 291-295 (1983).
146 Roberts, D. F. & Boyo, A. E. Abnormal haemoglobins in childhood among the Yorba. *Human Biology* **34**, 20-37 (1962).
147 Roberts, D. F. & Lehmann, H. A search for abnormal haemoglobins in some southern Sudanese peoples. *Br Med J* **1**, 519-521 (1955).
148 Saha, N. & Patgunarajah, N. Phenotypic and quantitative relationship of red cell acid phosphatase with haemoglobin, haptoglobin, and G6PD phenotypes. *Journal of medical genetics* **18**, 271-275 (1981).
149 Salzano, F. M. Incidence, effects, and management of sickle cell disease in Brazil. *The American journal of pediatric hematology/oncology* **7**, 240-244 (1985).
150 Samuel, A. P., Saha, N., Omer, A. & Hoffbrand, A. V. Quantitative expression of G6PD activity of different phenotypes of G6PD and haemoglobin in a Sudanese population. *Human Heredity* **31**, 110-115 (1981).
151 Sansarricq, H., Marill, G., Portier, A. & Cabannes, R. Les hemoglobinopathies en Haute Volta. *Sangre* **30**, 503-511 (1959).
152 Schedlbauer, L. M. & Pass, K. A. Cellulose acetate/citrate agar electrophoresis of filter paper hemolysates from heel stick. *Pediatrics* **83**, 839-842 (1989).
153 Schneider, R. G., Haggard, M. E., Gustavson, L. P., Brimhall, B. & Jones, R. T. Genetic haemoglobin abnormalities in about 9000 Black and 7000 White newborns; haemoglobin F Dickinson (Agamma97His-Arg), a new variant. *British Journal of Haematology* **28**, 515-524 (1974).

154 Silva Wdos, S., Lastra, A., de Oliveira, S. F., Klautau-Guimaraes, N. & Grisolia, C. K. Avaliacao da cobertura do programa de triagem neonatal de hemoglobinopatias em populacoes do Reconcavo Baiano, Brasil. *Cadernos de saude publica / Ministerio da Saude, Fundacao Oswaldo Cruz, Escola Nacional de Saude Publica* **22**, 2561-2566 (2006).
155 Simpore, J. *et al.* Glucose-6-phosphate dehydrogenase deficiency and sickle cell disease in Burkina Faso. *Pakistan Journal of Biological Sciences* **10**, 409-414 (2007).
156 Sokhna, C. S., Rogier, C., Dieye, A. & Trape, J. F. Host factors affecting the delay of reappearance of *Plasmodium falciparum* after radical treatment among a semi-immune population exposed to intense perennial transmission. *The American journal of tropical medicine and hygiene* **62**, 266-270 (2000).
157 Sommer, C. K., Goldbeck, A. S., Wagner, S. C. & Castro, S. M. Triagem neonatal para hemoglobinopatias: experiencia de um ano na rede de saude publica do Rio Grande do Sul, Brasil. *Cadernos de saude publica / Ministerio da Saude, Fundacao Oswaldo Cruz, Escola Nacional de Saude Publica* **22**, 1709-1714 (2006).
158 Spedini, G., Fuciarelli, M. & Rickards, O. Blood polymorphism frequencies in the Tofinu, the "Water Men" of Southern Benin. *Anthropologischer Anzeiger; Bericht uber die biologisch-anthropologische Literatur* **38**, 121-130 (1980).
159 Spivak, V. A., Sou, A. & Lutsenko, I. N. Rasprostranenie anomal'nykh gemoglobinov S i C v Gvineiskoi respublike. *Genetika* **28**, 159-165 (1992).
160 Sukumaran, P. K., Sanghvi, L. D. & Vyas, G. N. Sickle cell trait in some tribes of western India. *Current Science* **25**, 290-291 (1956).
161 Suzuki, A. *et al.* The distribution of hereditary erythrocytic disorders associated with malaria, in a lowland area of Nepal: a micro-epidemiological study. *Annals of Tropical Medicine and Parasitology* **101**, 113-122, doi:Doi 10.1179/136485907x154539 (2007).
162 Trabuchet, G., Dahmane, M. & Benabadji, M. Hémoglobines anormales en Algérie. *Semaine des Hôpitaux* **53**, 879-881 (1977).
163 Trincao, C. Hemoglobinas anormals nos territoris Portuguessas. *Boletim Clínico dos Hospitais Civis de Lisboa* **21**, 813-827 (1957).
164 Tshilolo, L. *et al.* Dépistage néonatal des hémoglobinopathies dans la région bruxelloise. *Revue medicale de Bruxelles* **18**, 70-73 (1997).
165 Van der Dijs, F. P. L. *et al.* Screening cord blood for hemoglobinopathies and thalassemia by HPLC. *Clinical Chemistry* **38**, 1864-1869 (1992).
166 van Heyningen, A. M. *et al.* Estimated incidence of sickle-cell disease in Aruba and St. Maarten suggests cost-effectiveness of a universal screening programme for St. Maarten. *West Indian Med J* **58**, 301-304 (2009).
167 Vandepitte, J. & Dherte, P. Enquête sur les hémoglobines anormales à Stanleyville. *Annales de la Societe Belge de Medecine Tropicale* **39**, 711-715 (1959).
168 Vandepitte, J. & Motulsky, A. G. Abnormal haemoglobins in the Kasai province of the Belgian Congo. *Nature* **177**, 757 (1956).
169 Vella, F. Hemoglobin variants in Saskatchewan. *Clinical Biochemistry* **1**, 118-134 (1967).
170 Vella, F. The human hemoglobin variants in Canada. *Clinical Biochemistry* **8**, 341-352 (1975).
171 Walters, J. H. & Lehmann, H. Distribution of the S and C haemoglobin variants in two Nigerian communities. *Trans R Soc Trop Med Hyg* **50**, 204-208 (1956).
172 Weatherall, D. J. Data for Sri Lanka. *Personal communication* (2010).
173 Yawson, G. & Marbell, E. C. Abnormal haemoglobins in 4000 blood donors at Korle Bu. *Ghana Medical Journal* **12**, 22-26 (1973).
174 Yorke, D. *et al.* Newborn screening for sickle cell and other hemoglobinopathies: a Canadian pilot study. *Clinical and investigative medicine* **15**, 376-383 (1992).
